# Comprehensive characterization of the embryonic factor LEUTX

**DOI:** 10.1016/j.isci.2023.106172

**Published:** 2023-02-09

**Authors:** Lisa Gawriyski, Eeva-Mari Jouhilahti, Masahito Yoshihara, Liangru Fei, Jere Weltner, Tomi T. Airenne, Ras Trokovic, Shruti Bhagat, Mari H. Tervaniemi, Yasuhiro Murakawa, Kari Salokas, Xiaonan Liu, Sini Miettinen, Thomas R. Bürglin, Biswajyoti Sahu, Timo Otonkoski, Mark S. Johnson, Shintaro Katayama, Markku Varjosalo, Juha Kere

**Affiliations:** 1 Stem Cells and Metabolism Research Program, University of Helsinki, 00290 Helsinki, Finland; 2 Institute of Biotechnology, University of Helsinki, 00790 Helsinki, Finland; 3 Folkhälsan Research Center, 00290 Helsinki, Finland; 4 Department of Biosciences and Nutrition, Karolinska Institutet, 14183 Huddinge, Sweden; 5 Applied Tumor Genomics Program, Research Programs Unit, Faculty of Medicine, University of Helsinki, 00290 Helsinki, Finland; 6 Department of Clinical Science, Intervention and Technology, Karolinska Institutet, 14186 Stockholm, Sweden; 7 Division of Obstetrics and Gynecology, Karolinska Universitetssjukhuset, 14186 Stockholm, Sweden; 8 Structural Bioinformatics Laboratory and InFLAMES Research Flagship Center, Biochemistry, Faculty of Science and Engineering, Åbo Akademi University, Turku, Finland; 9 RIKEN Center for Integrative Medical Sciences, Yokohama, Japan; 10 Institute for the Advanced Study of Human Biology, Kyoto University, Kyoto, Japan; 11 Department of Medical Systems Genomics, Graduate School of Medicine, Kyoto University, Kyoto, Japan; 12 IFOM-ETS, Milan, Italy; 13 Department of Biomedicine, University of Basel, Basel, Switzerland; 14 Centre for Molecular Medicine Norway (NCMM), University of Oslo, 0349 Oslo, Norway; 15 Children’s Hospital, Helsinki University Hospital and University of Helsinki, 00290 Helsinki, Finland

**Keywords:** Molecular biology, Omics, Reproductive medicine

## Abstract

The paired-like homeobox transcription factor LEUTX is expressed in human preimplantation embryos between the 4- and 8-cell stages, and then silenced in somatic tissues. To characterize the function of LEUTX, we performed a multiomic characterization of LEUTX using two proteomics methods and three genome-wide sequencing approaches. Our results show that LEUTX stably interacts with the EP300 and CBP histone acetyltransferases through its 9 amino acid transactivation domain (9aaTAD), as mutation of this domain abolishes the interactions. LEUTX targets genomic cis-regulatory sequences that overlap with repetitive elements, and through these elements it is suggested to regulate the expression of its downstream genes. We find LEUTX to be a transcriptional activator, upregulating several genes linked to preimplantation development as well as 8-cell-like markers, such as DPPA3 and ZNF280A. Our results support a role for LEUTX in preimplantation development as an enhancer binding protein and as a potent transcriptional activator.

## Highlights

• LEUTX directly interacts with EP300 and CBP
• The interaction with EP300 and CBP is lost when a 9aaTAD is deleted
• LEUTX binds and upregulates developmentally relevant enhancer and promoter regions
• LEUTX induction leads to activation of 8-cell-like expression marker genes

## Graphical abstract

**Figure:**
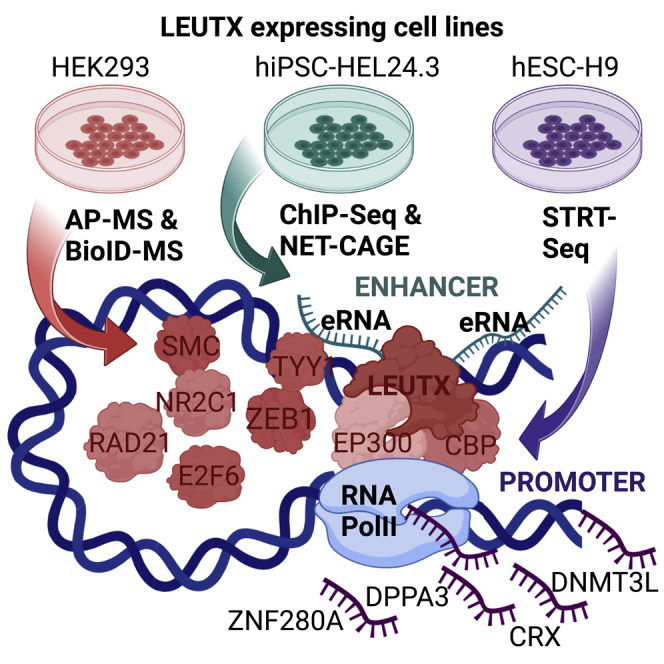

## Introduction

Human embryonic genome activation (EGA) is characterized by upregulation of a set of specific transcription factors and genomic repeat elements.^1^^,^^2^^,^^3^^,^^4^ One of the key EGA factors, DUX4, is expressed briefly in the zygote^5^^,^^6^^,^^7^^,^^8^ and drives the expression of non-coding repeat elements and its downstream genes, including *LEUTX*.^9^^,^^10^*LEUTX* is expressed at the 4- and 8-cell stages, and it is downregulated by the morula stage.^1^^,^^3^^,^^4^^,^^8^ However, there is mounting evidence that *Dux* or its regulators *Dppa2* and *Dppa4* are not essential for mouse preimplantation development and thus some authors have questioned whether *LEUTX* as a target of human *DUX4* is essential for EGA.^11^^,^^12^^,^^13^ On the other hand, we found that mutation frequencies in *LEUTX* are lower than the average of all human protein coding genes, suggesting that *LEUTX* is relatively constrained in human individuals. In 7 large human genotype resources, not a single individual with two deleterious variants of *LEUTX* were discovered.^14^ Recently Zou et al.^15^ found LEUTX knockdown to have a minor effect on EGA. These results motivate further study of the potential role of *LEUTX* in embryonic development.

LEUTX is a paired-like (PRDL) transcription factor (TF)^16^ with a complete functional K50 homeodomain.^1^^,^^14^^,^^17^ It is thought to have arisen by tandem gene duplication and subsequent asymmetric sequence evolution from the cone-rod homeobox gene *CRX* (*OTX5*) from the Otx gene family.^18^^,^^19^ Additional genes in this family such as *ARGFX*, *DPRX*, *TPRX1*, and *TPRX2* are all expressed during human preimplantation development.^1^

In this study we present a comprehensive characterization of LEUTX using two different proteomics methods and three different genome-wide approaches. We performed affinity purification (APMS) and BioID-MS using stable Flip-In T-REx 293 cell lines expressing MAC-tagged LEUTX,^20^ and RNA sequencing (STRT-Seq on bulk-RNA, modified from single-cell tagged reverse transcription sequencing protocol),^21^^,^^22^ native elongating transcript-cap analysis of gene expression (NET-CAGE),^23^ and LEUTX targeted chromatin immunoprecipitation sequencing (ChIP-Seq)^24^ using human pluripotent stem cells (hPSCs) with doxycycline inducible *LEUTX*. Because of ethical reasons and the scarcity of biological material available, it is impractical to study the very first steps of human development, including functions of the EGA-associated genes, directly in human embryos. Although the cell lines used in this study do not fully mimic the regular context of cleavage stage embryo, they represent practical means to collect large amounts of LEUTX expressing cells required for high throughput experiments. Our results indicate LEUTX as a potent chromatin modifier, which interacts stably with histone acetylases EP300 and CBP^25^ and dynamically with varied chromatin modifying complexes. We show that LEUTX binds repetitive elements, regulatory sequences including promoters and enhancers, and regulates the expression of pluripotency-associated factors.

## Results

### Functional domains of LEUTX

**Figure 1:**
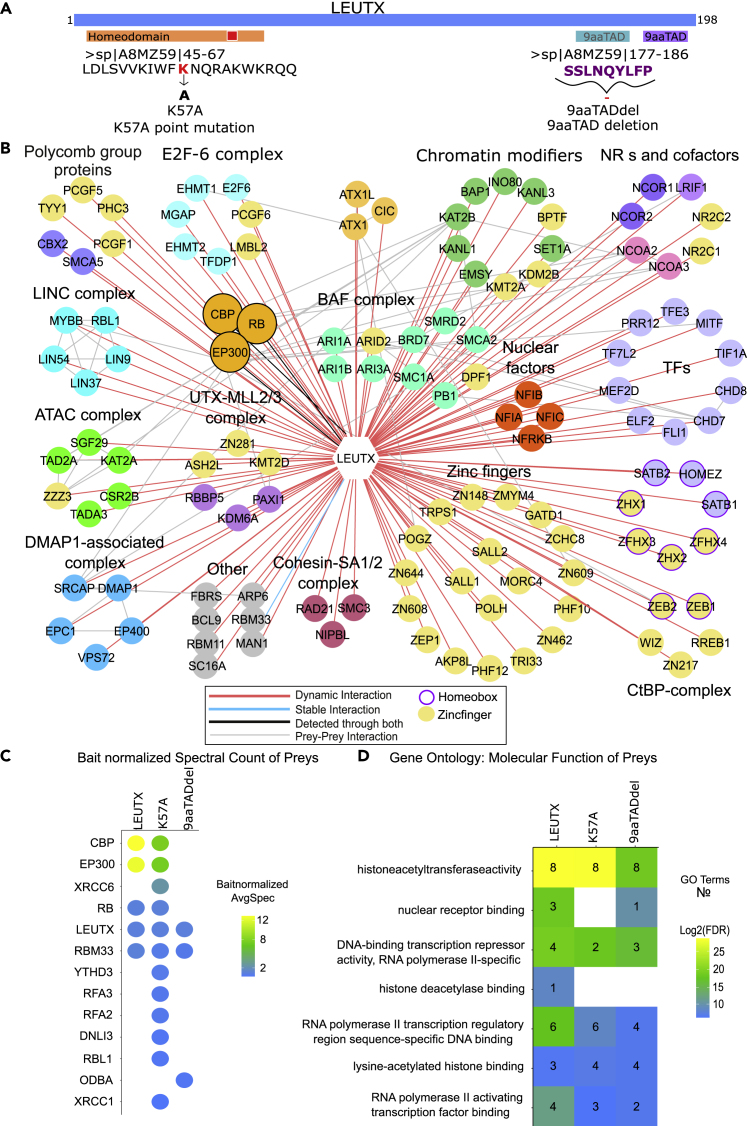
LEUTX protein-protein interactions (A) Overview of known functional domains of LEUTX. The N-terminal homeodomain is highlighted in orange with a K57A point mutation in red. ^178^SSLNQYLFP^186^ 9aaTAD deletion is marked as purple in the far C-terminal region. Another computationally predicted, but less conserved, 9aaTAD is highlighted in teal. Figure shows the amino acid sequences modified in this study. See also Figure S1 and Table S13. (B) LEUTX stable and dynamic interactome. Dynamic BioID-MS interactions are indicated by red lines (124), stable AP-MS interactions by blue line and interactions detected by both by black lines and border, node highlighted in orange. Names are UniProtKB entry names (protein identification codes). Known Prey-Prey interactions in the iRef database are depicted in grey lines. Zinc finger proteins are highlighted in mustard yellow node color; altogether 47 preys were zinc finger proteins. Homeobox domain proteins are highlighted with purple border. Chromatin modifying complexes displayed by name are significantly enriched in the interactome (FDR < 0.05). See also Figure S2, Table S1 for complete results and Table S2 for CORUM Complex enrichment analysis results. See Table S3 for expression of identified interactors in embryonic transcriptomics dataset. (C) LEUTX and its mutants’ stable AP-MS interactions. Bait Normalized Spectral Count of AP-MS interactions in LEUTX and two functional mutants. Color depicts bait normalized spectral count (Average spectral count of Prey/Average spectral count of Bait). (D) GO Molecular Function heatmap of interactors between LEUTX and mutants. Gene Ontology terms reduced to the highest order term by using redundancy based on semantic similarity; number of combined terms depicted in black numbers and Log2 FDR indicated in color (FDR < 0.05). See also Figure S2B for GO: Cellular Compartment enrichment.

LEUTX has a PRDL homeodomain and two predicted nine-amino-acid transactivation domains (9aaTADs), both located near the C-terminal end of LEUTX (Figure 1A).^14^ The ^178^SSLNQYLFP^186^ 9aaTAD was found to be the more conserved of the two and as such is considered to be a putatively active interaction domain.^14^ The homeodomain recognizes DNA and in our previous study we demonstrated that the K57A mutation in the LEUTX homeodomain eliminates binding to the recognized DNA motif.^14^ 9aaTADs may directly interact with kinase-inducible (KIX) domains, highly conserved globular domains with three α-helices.^26^ The most well-known coactivators having KIX-domains include histone acetyltransferases CBP, EP300, and transcriptional coactivator MED15.^26^^,^^27^

To study the structural basis of LEUTX protein-protein interactions, we built a structural model of the predicted 9aaTAD motif (^178^SSLNQYLFP^186^) bound to the KIX domain of the CBP/p300 (Figures S1A and S1B; NMR structure, PDB code 2LXT ^28^). We compared our model to the known structural complex with a mixed-lineage leukemia (MLL) 9aaTAD sequence, and the model suggests conservation of key interactions (Figure S1C). To model the effect of the K57A mutation in the LEUTX homeodomain, we built a structural model of the homeodomain-DNA complex of LEUTX with the K57A mutation,^14^ which suggests that multiple binding interactions are lost (Figure S1D).

### LEUTX interacts with multiple chromatin modifying proteins and protein complexes

To study LEUTX protein-protein interactions, we performed mass spectrometry-based interactome analyses by two complementary methods, affinity purification (APMS) that reveals stable interactions and BioID-MS that reveals dynamic proximity labeled interactions captured over 24 h in HEK293 cells using the MAC-tag.^20^^,^^29^ As a negative control we used GFP tagged with a nuclear localization signal. We detected a total of high-confidence 129 protein-protein interactions for LEUTX, out of which 5 were detected by AP-MS and 124 by BioID-MS (Figure 1B, Table S1). LEUTX stably interacted with KIX-domain containing histone acetyltransferase EP300 and cofactor CBP (Figure 1C). EP300 and CBP are known to interact with each other and positively regulate transcription by catalyzing the active chromatin mark H3K27ac found in active enhancers.^25^^,^^30^^,^^31^ We confirmed that the key interactors EP300 and CBP and a well-known transcriptional coactivator MED15, are all expressed in the cleavage stage embryo (Figure S2A).^4^ LEUTX also interacts with cell cycle controller and histone modifier RB (Figure 1B) that is lowly expressed in the 4-cell embryo.^4^

By BioID-MS we detected dynamic interactions with several proteins that act as part of chromatin modifying complexes. According to the CORUM enrichment analysis, the most enriched (Fisher’s exact test, FDR < 0.05) complexes were the E2F-6 complex (7 interactions), the UTX-MLL2/3 complex (6), the ATAC complex (6) and the full multisubunit ACTR coactivator complex (Figure 1B, Table S2). The protein database CORUM lists multiple possible isoforms for these complexes, and it is thus not possible to distinguish the exact isoform from affinity purification data.^32^ GO-terms related to histone acetylation, regulation of transcription, and cell cycle progression are enriched among the LEUTX interactors (Figures 1D and S2B). A total of 56% of the LEUTX interactors were listed as having epigenetic function in the EpiFactors database,^33^ with the most typical known functions for these proteins related to histone modification (Figure S2C).

### 9aaTAD deletion eliminates interaction with EP300 unlike the K57A homeodomain mutation

Next, we investigated how the inactivation of the functional domains affects the interactome. Based on structural information we predicted that the ^178^SSLNQYLFP^186^ 9aaTAD deletion mutant would lose interaction with EP300 and CBP and that the K57A homeodomain mutant would lose binding affinity to DNA. To confirm, we performed a full interaction analysis on the two functional mutants: the K57A homeodomain mutant and the 9aaTAD deletion mutant. For the LEUTX 9aaTAD mutant the stable interaction with EP300, CBP and RB was lost (Figure 1C). RB does not contain a KIX-domain and in previous research, an interaction between EP300/CBP and RB has been shown.^34^ Because the affinity purification cannot reveal if RB was bound to EP300, CBP or LEUTX we cannot confirm the direct interaction between LEUTX and RB. The interactions with EP300 and CBP are still detected through BioID-MS for the 9aaTAD deletion mutant, but significantly weakened compared to the wild type (Student’s *t*-test, EP300 p-value = 3E-5, CBP p-value = 1E-6) (Figure S2D). The K57A mutant still maintained direct interaction with EP300, CBP and RB (Figure 1C). The ‘Nuclear Receptor Binding’ GO-term was lost for the K57A mutant but was maintained for the 9aaTAD deletion mutant (Figure 1D). The K57A mutant also lost interactions with other TFs and chromatin modifiers, and based on its interactome, it appeared displaced from the nuclear matrix (Figure S2B).

Altogether, we detected 149 unique high-confidence interactors for LEUTX and the domain mutants, of which the vast majority (98; 66%) are general factors detected on RNA level in all tissues in The Human Protein Atlas (Figure S2E).^35^ 129 (88%) of the unique interactors were found expressed in Yan et al. (2013)^4^ embryonic sequencing dataset between 2-cell and Morula stages with RPKM > 1 in at least one timepoint (Table S3). Most of the interactions detected for LEUTX and the mutants are shared in all (65; 44%) or detected in LEUTX and one of the mutants only (LEUTX and K57A: 16; 11%, LEUTX and 9aaTADdel: 23, 15%) (Figure S2F).

### LEUTX binds close to the interactors’ binding sites and regulates both enhancers and promoters

**Figure 2:**
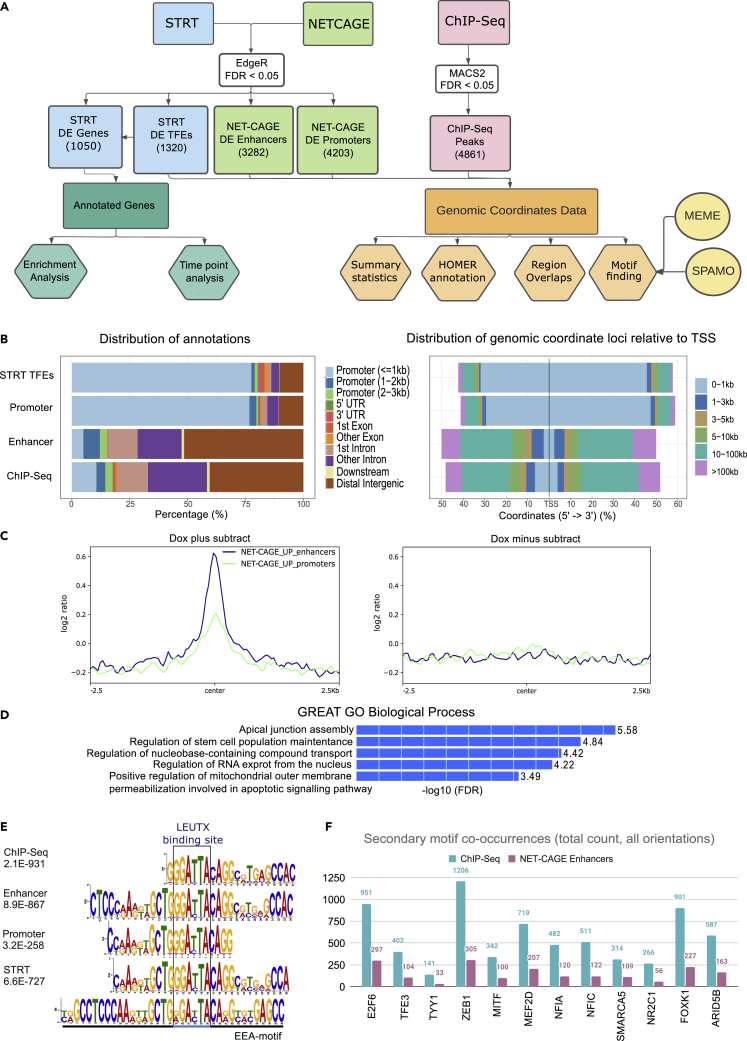
Genomics Overview (A) Overview of sequencing experiments and data analysis pipeline. Three different genome-wide analyses; modified STRT-Seq (blue), NET-CAGE (green) and ChIP-Seq (pink) produce complementary, but functionally different genomic coordinates. STRT-Seq also leads to traditional gene lists to analyze up- and downregulated terms and enrichment of biological functions. Multiple analyses are listed in hexagonal boxes and the motif finding tools in yellow circles. See also Figure S3 and Table S4 for statistically significantly upregulated NET-CAGE enhancer locations, Table S5 for statistically significantly upregulated NET-CAGE promoter locations, Table S6 for differential ChIP-Seq peaks. (B) Annotation of genomic regions. The annotation of genomic regions obtained through ChIPSeeker R-package using GENCODE annotations.^39^ Left panel shows the distribution of the annotations in percentages and right panel shows the distribution relative to TSS. STRT and NET-CAGE promoter peaks are enriched near annotated promoters while NET-CAGE enhancers and ChIP-Seq peaks are often located in intergenic or intronic locations. TSS = transcription start site. (C) ChIP-Seq peaks overlayed with NET-CAGE regulatory regions. Overlapping of LEUTX induced (Dox+ and Dox- sample) ChIP-Seq peaks to upregulated NET-CAGE promoter and enhancer regions. To produce this plot, genome is partitioned into bins of equal size, and then reads are counted per bin. Y-axis is the log2 ratio of number of NET-CAGE reads per bin between the Dox+ and Dox- subtracts of ChIP-Seq samples, whereas the x-axis is distance from center of ChIP-Seq peaks (bp). Upregulated NET-CAGE enhancers are shown in blue and upregulated NET-CAGE promoters are shown in green. (D) Genomic Regions Enrichment of Annotations Tool (GREAT) enrichment of LEUTX ChIP-Seq peaks. Top 5 GO terms for Biological Process enriched in GREAT enrichment analysis sorted by their FDR value. GREAT assigns Gene Ontology (GO) terms based on annotations of nearby genes. (E) Motif finding results. The top motifs identified through motif finding tool MEME in all genomics datasets overlaid over the previously identified EEA-motif. Expected-value (E-value) produced by the MEME tool listed in the figure. (1) ChIP-Seq top motif hit E-value = 2.1E-931, (2) NET-CAGE enhancer top motif hit E-value = 8.9E-867, (3) NET-CAGE promoter top third motif hit E-value = 3.2E-258, (4) STRT TFE motif top hit E-value 6.6E-727. The LEUTX binding site 5′-GGATTA-3′ is highlighted in blue. (F) Spatial Motif finding results. MEMESuite SpaMo motif finding tool was applied to search for motifs enriched proximal to the EEA-motif in our datasets. In total, we found 145 motifs that were significantly enriched proximally to the EEA-motif in all datasets (differentially upregulated NET-CAGE enhancers, NET-CAGE promoters and STRT TFEs, and ChIP-Seq peaks), out of which 12 were also detected through BioID-MS proteomics. Highlighted here are the number of total binding sites detected proximally to the EEA-motif (SpaMo output total) in key factors also detected through BioID-MS and protein-protein interaction complex enrichment analysis, in ChIP-Seq (teal) and NETCAGE-Enhancer (purple) sequence data. See Table S7 for complete results.

To study how LEUTX acts as a transcription factor, we performed three different types of complementary genome-wide analyses using the LEUTX-TetOn hPSCs (overviewed in Figure 2A and S3). Although hPSCs do not mimic the actual molecular context of the cleavage stage embryo, but rather several days later epiblast phase, they represent a feasible model to study EGA-associated genes using methods that require millions of cells. Recently developed methods to detect and enrich human 8-cell like cell populations among hESC or naive hPSC cultures provide a new tool for further characterization of human EGA-associated factors.^36^^,^^37^^,^^38^ The 8 cell-like cells, however, represent minor subpopulation among hPSC or naive hPSC cultures and are unstable with a tendency to convert back to later developmental stages and as such are not feasible for the production of large amounts of cells expressing transgenic gene of interest.

First, we applied the NET-CAGE method to detect 5′ ends of newly synthesized promoter RNAs and bidirectionally expressed enhancer RNAs (eRNA) that indicate active enhancer positions.^23^ Second, the modified STRT-Seq analysis, here performed on bulk-RNA, yielded Transcript Far 5′ Ends (TFEs) that were used to quantify gene expression.^21^ Finally, we performed LEUTX-targeted (HA) ChIP-Seq which produces genomic coordinate peaks reflecting genomic binding sites of LEUTX (Figure 2A). Together these methods provide us a global insight where LEUTX binds in the genome and how it regulates the expression of not only protein coding genes but also the regulatory genome such as enhancers and promoters.

NET-CAGE sequencing of HEL24.3 iPSCs expressing doxycycline inducible transgenic LEUTX identified 3282 differentially expressed (FDR < 0.05) enhancers and 4203 differentially expressed (FDR < 0.05) promoters, out of which 1990 and 2664 were upregulated respectively (logFC > 0) (Figures S4A and S4B, Tables S4 and S5). Next, we annotated the upregulated enhancers and promoters towards known GENCODE TSS regions and showed that the NET-CAGE promoters primarily annotated to proximal promoter regions, whereas the NET-CAGE enhancers mostly annotated to distal intergenic and intronic regions (Figure 2B).

The FANTOM5 consortium has identified ∼65 000 human transcribed enhancers by sequencing 1829 human samples.^40^^,^^41^ We compared our 1990 upregulated LEUTX induced enhancers to previously published enhancers and found that only 657 were included in the FANTOM5 project, with 1333 thus being novel.^42^ We also compared the LEUTX induced enhancers to those upregulated by DUX4,^9^ and found 269 overlapping upregulated enhancers. We further compared the identified genomic regions to publicly available regulatory region datasets to further annotate their function. 160 upregulated enhancers overlapped with known super-enhancer locations in dbSuper H1 dataset (23% of H1 super enhancers).^43^

To study the effects of LEUTX expression at physiological level, we generated a hESC cell line conditionally expressing dCas9-VP192 activator together with guides targeting LEUTX promoter and enhancers identified by Vuoristo et al. (2022)^9^ (Figure S5). The activation of the enhancers that result in induction of LEUTX may, however, also affect the genomic region surrounding the LEUTX locus. We analyzed transcriptome effects by STRT at 24 h, 48 h, and 72 h after LEUTX induction, comparing them to no-induction controls. We found differential expression (FDR < 0.05) of 1050 genes in at least one time point. Similar to the NET-CAGE promoter dataset, STRT primarily detects the 5′ ends of the transcripts characterizing the promoter level expression and GENCODE annotations were comparable to NET-CAGE promoter annotations (Figure 2B).

Next, we identified 4861 differential (FDR < 0.05) ChIP-Seq peaks using HEL24.3 iPSCs expressing doxycycline inducible transgenic HA-tagged LEUTX. The ChIP-Seq peaks mapped mostly to distal intergenic and intronic regions (Figure 2B, Table S6). We performed region overlap analysis to identify whether ChIP-Seq regions overlap with the NET-CAGE promoter and enhancer data.

The data showed that LEUTX binding sites overlap with both upregulated promoters and enhancers rather than downregulated ones with higher incidence with upregulated enhancers (82 ChIP-Seq peaks directly overlap upregulated NET-CAGE promoters, 308 ChIP-Seq peaks directly overlap upregulated NET-CAGE enhancers) (Figures 2C, S4C, and S4D). Genomic Regions Enrichment of Annotations Tool (GREAT) analysis of ChIP-Seq peaks showed enrichment of the terms apical junction assembly, regulation of stem cell population maintenance and nucleobase/RNA transport (Figure 2D).

To explore the function of the detected binding sites, we then compared differential LEUTX ChIP-Seq peaks with preimplantation embryo Assay for Transposase-Accessible Chromatin using sequencing (ATAC-seq) data,^44^ and found that LEUTX preferentially binds accessible chromatin regions identified in the 8-cell stage, as compared to 2-cell, 4-cell, and ICM (Figure S6A). These comparisons suggest that LEUTX regulates a set of genomic regions that are accessible during embryonic development.

Furthermore, we compared our data to publicly available ENCODE TF ChIP-Seq datasets (Figures S6B and S6C). We found that even with differences in cell lines, batch effects, and other experimental differences LEUTX ChIP-Seq peaks were often proximal with known EP300 binding sites particularly in H1 cell line data, in comparison to cancer cell lines (Figure S6B). Of interest, binding sites for RAD21 and SMC, components of the cohesin complex identified through our BioID-MS, were also often proximal to LEUTX binding sites (Figure S6C).

In our previous study, LEUTX was found to bind a 36 bp motif enriched in promoters of genes involved in EGA (EEA-motif).^1^^,^^14^^,^^45^ Motif analysis of all genomic datasets included in the current study showed strong enrichment of this motif, with the whole or partial EEA-motif found in every dataset and as one of the top three highest-confidence motifs (Figure 2E). In ChIP-Seq (E-value = 2.1E-931), upregulated NET-CAGE enhancer (E-value = 8.9E-867), and STRT TFE (E-value = 6.6E-727) data it was the top hit, and in upregulated NET-CAGE promoters (E-value = 3.2E-258) it was the third motif hit sorted by E-value (Figure 2E). Further, using the MEMESuite tool SpaMo we analyzed which motifs were enriched proximal to the EEA-motif in the genomic coordinates implicated by our data (NET-CAGE Enhancers, NET-CAGE Promoters, ChIP-Seq peaks, and STRT TFEs). We found 144 motifs that were significantly enriched in all datasets, out of which 12 were detected through proteomics (Table S7). Most notably, E2F6 (E2F6 Complex), TYY1 (Polycomb repressive complex 1), ZEB1 (CtBP complex), and SMARCA5 (BAF-complex) binding sites were enriched proximal to LEUTX binding sites and detected as protein-protein interactors of LEUTX (Figure 2F).

### LEUTX binds to repetitive elements and non-coding RNA transcription start sites

**Figure 3:**
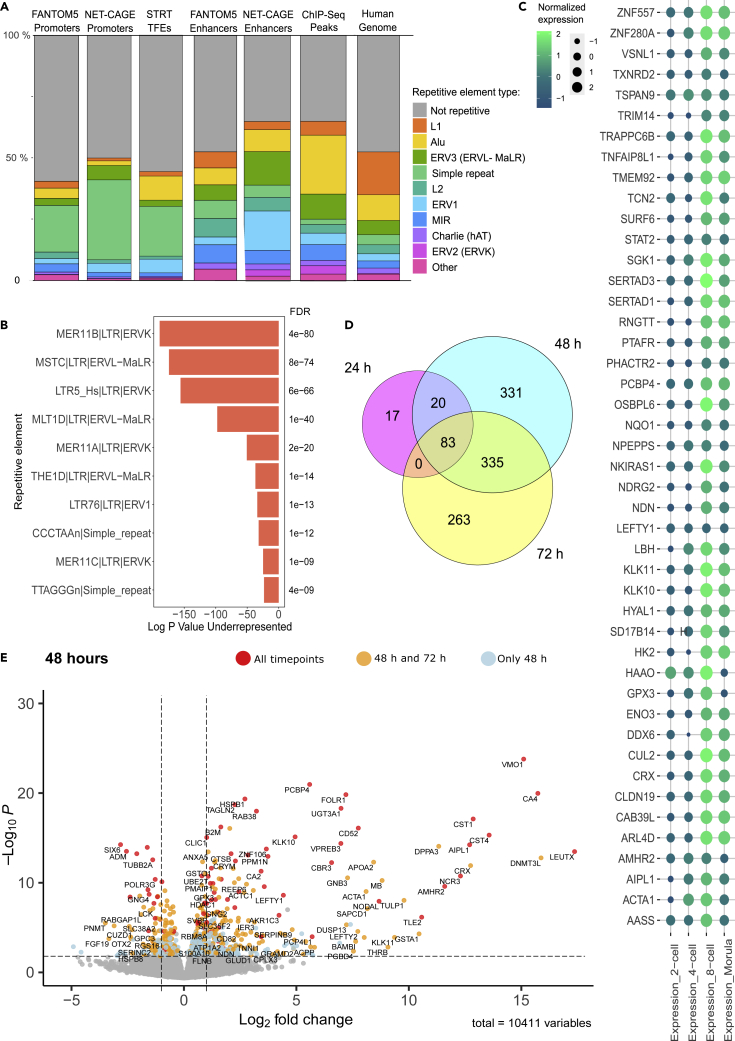
LEUTX binds repetitive elements and regulates EGA-associated genes (A) The proportion of different repetitive elements in all our genomic datasets, compared to their frequency in the human genome and FANTOM5 datasets. FANTOM5 Promoters and FANTOM5 Enhancers refer to the FANTOM5 CAGE Promoters and Enhancers datasets from the FANTOM5 project, NET-CAGE Promoters, NET-CAGE Enhancers, STRT TFEs and ChIP-Seq peaks refer to the datasets introduced in the current study. Y-axis shows the cumulative proportions in percentages. See also Table S8. (B) Most common single repetitive elements identified overlapping LEUTX ChIP-Seq binding sites. HOMER repetitive element enrichment analysis for the ChIP-Seq peaks is compared to genomic frequency to produce estimates of under- or over enrichment. Overrepresentation is shown as red bars growing in the negative direction (Log PValue Underrepresented). Also shown is a multiple analysis corrected p-value under the FDR column. See also Table S9. (C) Expression of key LEUTX targets in preimplantation embryo datasets. The plot shows genes that are differentially expressed in our STRT-Seq data and also expressed in human cleavage stage embryos according to both Yan et al. (2013)^4^ and Liu et al. (2018).^8^ Shown targets peak in expression at the 8-cell stage, which coincides with biological expression of LEUTX in cleavage stage embryos. The intensity of the color and size of the circles indicate the normalized expression with values from Liu et al. (2018)^8^ of genes that were found expressed in cleavage stage embryos in both Yan et al. (2013)^4^ and Liu et al. (2018).^8^ (D) The number of differentially expressed genes in different time points in STRT-Seq data. Venn diagram showing the overlap between DE genes at timepoints 24h, 48h and 72h following LEUTX induction. (E) Volcano plot of differentially expressed genes after the LEUTX induction at time point 48 h compared to no-dox controls. Genes that are differentially expressed in all time points (24h, 48h & 72h) are shown in red and those shared in 48h and 72h shown in orange, both labelled with gene symbols. Genes that are differentially expressed in 48 h timepoint only are shown in blue. See also Tables S10 and S11.

Because many of the identified regulatory regions (STRT-Seq TFEs, NET-CAGE identified promoters and enhancers) or those bound by LEUTX (ChIP-Seq peaks) were far away from annotated promoter or TSS regions and as the EEA-motif was enriched among all datasets, we investigated whether the genomic coordinates from our different datasets overlapped with repetitive elements. In the STRT-Seq data 614 unique TFEs (45% of all TFEs) overlapped with repetitive elements. 1334 upregulated NET-CAGE promoters overlapped 1733 repetitive elements (50% of all promoters), and 1299 uniquely upregulated NET-CAGE enhancers overlapped with 1732 repetitive elements (65% of all enhancers). In the ChIP-Seq data, 3160 differentially expressed unique peaks directly overlapped with 4359 known repetitive elements (65% of peaks). Next, we compared the repetitive element overlap frequencies in STRT-Seq TFEs and LEUTX-driven NET-CAGE promoters to that of FANTOM5 promoters (Figure 3A). The results show that there was more repetitive element overlap in LEUTX-driven NET-CAGE promoters than in FANTOM5 promoters (Chi-squared test p < 2.2E-16) and microsatellites and simple repeats were overrepresented particularly in upregulated STRT TFEs and NET-CAGE promoters (Figure S7A) while common LINE-L1 elements were underrepresented.

We also compared the observed overlap frequencies of upregulated LEUTX-driven NET-CAGE enhancers to FANTOM5 enhancers and found that LEUTX driven enhancers had relatively more overlap with repetitive elements (Chi-squared test p < 2.2E-16) and particularly more ERV1 and MaLR elements (Chi-squared test, ERV1 p = 2.29E-206, MaLR p = 7.18E-39, Figure 3A, Table S8). HERVH (ERV1) elements were particularly overrepresented in upregulated NET-CAGE enhancers (Figure S7A). In all cases, the most common LINE-L1 elements were underrepresented (Figure S7A, Table S8).

LEUTX binding sites revealed through ChIP-Seq, showed notable binding to Alu elements (24% of all identified binding sites and 36.9% of all overlapping repetitive elements); the most enriched overlapping Alu element compared to genomic frequency was AluJb (Figures S7B and S7C and Table S9). This enrichment is in agreement with the earlier finding of the 36 bp EEA motif in Alu elements.^1^ However, we do not detect significant LEUTX binding to MLT2A1 or LTR12C which have been recently described as highly accessible in both human 8 cell embryos and 8CLC model.^36^ Most enriched repetitive elements overlapping LEUTX binding sites compared to genomic frequency are MER11B (ERVK), MSTC (ERVL-MaLR), LTR5_Hs (ERVK), MLT1D (ERVL-MaLR), and MER11A (ERVK) (Figures 3B and S7A, Table S9). To conclude, our data suggests that many LEUTX-associated regulatory regions overlap with repetitive elements. However, the data provides only indirect evidence that LEUTX itself regulates transcription through binding to the repetitive elements.

### *LEUTX* expression leads to a cascade of transcriptional activation

Next, we performed deeper analysis to understand the transcriptional effects of endogenous LEUTX activation in a hPSC model. We analyzed transcriptome effects by STRT at 24 h, 48 h, and 72 h after LEUTX induction, comparing them to non-induction controls. To address the validity of our hPSC model, we compared the differentially expressed genes by LEUTX activation to those expressed by human cleavage stage embryos. We found that out of 1048 genes regulated by LEUTX in at least one time point (FDR < 0.05), 836 were detected expressed in human embryos by Yan et al. (2013)^4^ single-cell data between the oocyte and morula stages (RPKM>1 in at least one embryonic cell stage) and 427 were found expressed between the 2-cell and morula stages by Liu et al. (2019)^8^ (Figure S8A). Even though hPSCs do not fully mimic the molecular context of cleavage stage embryos where LEUTX is naturally expressed, the majority of LEUTX targets detected here in hPSCs are active also in the relevant cell stages. 124 of upregulated LEUTX targets were found in both Yan et al. (2013)^4^ and Liu et al. (2019)^8^ studies, and 45 genes detected in both studies peak in expression at the 8-cell stage in Liu et al. (2019)^8^ (Figure 3C). We further compared our differentially expressed gene lists to the recently published 8-cell-like datasets,^36^^,^^37^^,^^38^ and confirmed LEUTX expression in 8-cell-like cell population but not in primed stem cells.

The number of differentially expressed genes increased notably from 24 h to 48 and 72 h (Figure 3D, Table S10). The 48- and 72-h timepoints are expected to include both primary and secondary targets of LEUTX. 83 genes were differentially expressed at all time points, of which the most upregulated genes were *CA4*, *VMO1*, *NCR3*, *CST4*, *TLE2*, *AIPL1*, and *CST1* (all with average log2FC > 10) whereas the most downregulated genes were *C9orf135* and *SIX6* (average log2FC < -2) (Table S10). Overall, LEUTX induction caused notable upregulation (average log2FC > 1) of 342 genes and downregulation (average log2FC < -1) of 162 genes, emphasizing its role as a transcriptional activator (Figure S8B, Table S10). This is in line with previous findings characterizing LEUTX as a transcriptional activator.^17^ LEUTX induction led to differential expression of several other TFs, such as upregulation of *TLE2*, *KLF6*, *ELF3*, *CRX*, *DPPA3* and downregulation of *SIX6*, *MYC*, *OTX2*, and *FOXH1* (Figures 3E, S8C, and S8D, Table S10). *DPPA3* (aka *Stella*) was strongly upregulated at 48 h and 72 h (average log2FC > 11). *DPPA3* has been linked to maintenance of methylation of developmental promoters in the early embryo and as a naive pluripotency marker.^46^^,^^47^^,^^48^^,^^49^

LEUTX induction also altered the expression of 14 epigenetic modifiers, most notably *DNMT3L*. *DNMT3L*, a catalytically inactive DNA methyltransferase was strongly upregulated at 48 h and 72 h (Figures 3E, S8C, and S8D, Table S10). *DNMT3L* is linked to *de novo* DNA methylation during EGA.^8^*HDAC1* was upregulated at all time points (average log2FC 1.1), whereas *PHC1*, a component of the Polycomb repressive complex was downregulated at all time points (average log2FC -1.1) (Table S10). Of interest, LEUTX induction led to differential regulation of the expression of at least 29 known pluripotency factors.^50^^,^^51^ Out of these, we found upregulation of *FGF13*, and naive pluripotency markers *DPPA3* and *NODAL*, and its antagonists *LEFTY1* as well as *LEFTY2*, and downregulation of *OTX2*, *CRABP1*, *PRDM14*, *C9orf135*, *NTS*, and *TDGF1* (Table S10). Further, LEUTX induction led to downregulation of primed pluripotency markers DUSP6 (average log2FC –0.9), KLHL4 (-1.1), ZDHHC22 (-1.6), and NEFM (72 h only, -2.6).^52^ LEUTX induction led to differential expression of 33 different cell signaling and receptor genes (Table S10).

Further, we compared the list of upregulated genes in this study to the upregulated LEUTX targets in our previous study,^17^ and found 205 LEUTX targets upregulated in both datasets (Table S11). The common targets included *CA4*, *VMO1*, *CST1*, *DPPA3*, *SGK1* and *NODAL* which were among the most upregulated in this study.

Finally, our STRT-Seq data revealed strong upregulation of *CRX* (log2FC 48h: 12.7, 72h: 10.9) and downregulation *CRX*’s ancestral family member *OTX2* (log2FC 48h: -2.3, 72h: -1.6) (Figures 3E, S8C, and S8D). We found several LEUTX driven genomic locations in the *CRX* genomic locus: one TFE, one NET-CAGE promoter, one putative enhancer, and two ChIP-Seq peaks directly on the *CRX* promoter, and we found one putative intergenic enhancer (Figure S9A). Further, at the adjacent genomic locations, we detected two distal upstream enhancers that were upstream of *TPRX1*, two downstream enhancers, upstream of *TPRX2* and three ChIP-Seq peaks downstream of *CRX*. *CRX* has been shown to be upregulated at the 8-cell stage of human development.^8^

**Figure 4:**
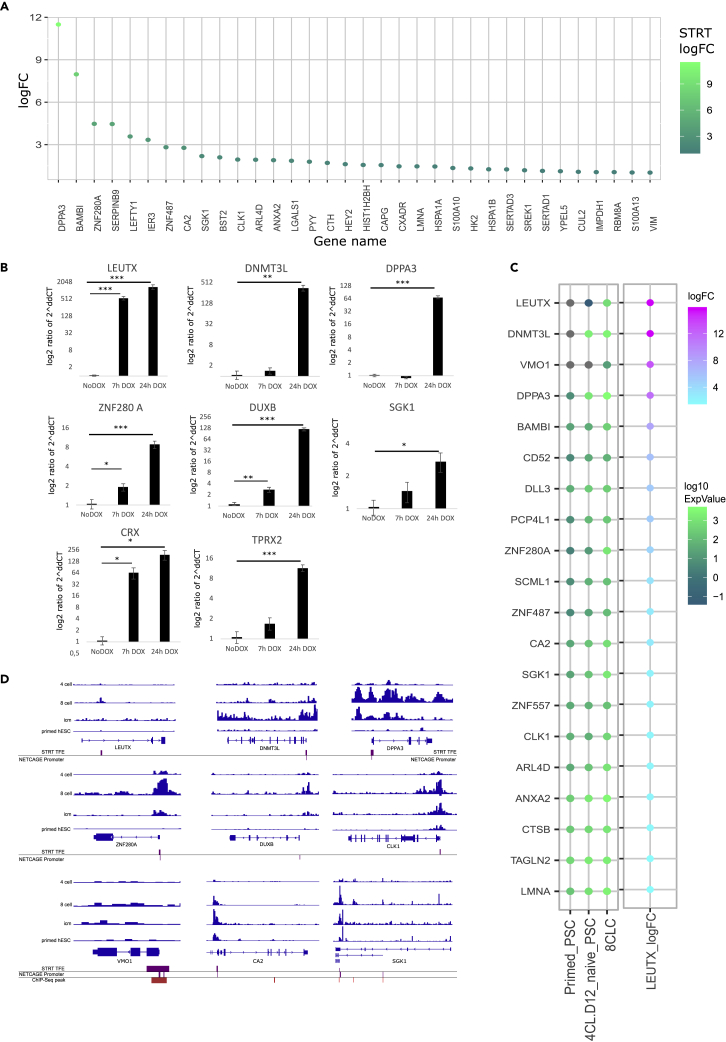
Comparison of LEUTX targets and 8CLC markers (A) Differentially expressed genes in LEUTX STRT and 8CLC data. 34 genes were found to be both differentially expressed and upregulated in our STRT-Seq data and in at least two of the following datasets: 8CLC markers in Taubenschmid-Stowers et al. (2022),^37^ DEG in 8CLC compared to non-8CLC in Mazid et al. (2022),^36^ or as iBM genes in Yoshihara et al. (2022).^38^ The intensity of the color reflects the logFC of the gene in LEUTX STRT-Seq. (B) Validation of target genes up-regulated by LEUTX according to our STRT-Seq data. The target gene expression was measured by qRT-PCR from independent transgenic Tet-On LEUTX cell line at time points 7h and 24h and compared to the mean expression without induction (no dox). Y-axis shows the log10 ratio of -2ˆddCT. n = 4 inductions. See also Table S12. (C) Expression of key LEUTX targets in 8CLC dataset. The plot shows genes that are differentially expressed in our STRT-Seq data and also peak in expression in the 8CLC stage in Mazid et al. (2022)^36^ hub genes. The intensity of the green color reflects the logarithmic relative expression of hub genes in three cell states from Mazid et al. (2022).^36^ The intensity of the cyan to magenta color is the logFC of the genes in our STRT-Seq. Total number of hub genes found differentially expressed in our STRT-Seq data is 277, figure is filtered to show top 20 genes with highest logFC in LEUTX STRT-Seq. (D) Chromatin state changes between embryonic cell stages of LEUTX targets. The embryonic ATAC-Seq data from Wu et al. (2018)^44^ from 4-cell, 8-cell and ICM embryos and primed hESCs over key gene regions identified through our datasets. Genomic locations of differentially expressed STRT-Seq TFEs and NET-CAGE promoters shown in purple, and differential ChIP-Seq peaks shown in red, relative to their position to key genes.

We validated the *CRX* upregulation upon *LEUTX* expression by qRT-PCR in independent transgenic doxycycline inducible cell line (Figure 4B). To test the functionality of putative *CRX* enhancer-like region, we used CRISPR activation by dCas9-VP192^45^ in combination with guide RNA (gRNA) pools to target the promoter and putative enhancer-like regions in HEK293 cells (Figures S9B and S9C). Activation of the *CRX* enhancer region upstream of the promoter but not the intergenic one led to upregulation of *CRX* expression level compared to the non-transfected control (Figure S9B). Furthermore, co-transfection of the pool of *CRX* enhancer targeting guides together with the *CRX* promoter targeting guides led to increased expression level compared to promoter activation only. This finding supports the functionality of LEUTX-activated putative *CRX* enhancer.

### LEUTX contributes to the expression of 8-cell like expression markers

Recently developed 8-cell-like cell (8CLC) models represent hESCs or human naive PSCs guided to transcriptionally resemble the human 8-cell embryo. Three recent studies identify a number of 8CLC signature and marker genes.^36^^,^^37^^,^^38^ We compared the differentially expressed genes from our STRT-Seq to the identified 8CLC signature genes from these papers. Combined, all 1048 differentially expressed genes from LEUTX STRT-Seq match 377 genes identified in at least one of these papers (Table S10). Altogether, 34 genes were identified upregulated (logFC > 1) in our STRT-Seq and in at least two of the studied 8CLC datasets (Figure 4A), and 8 genes, DPPA3, CA2, CLK1, ARL4D, HK2, HSPA1B, SERTAD1, and PDCL3, are upregulated after LEUTX expression in our data and are listed in all four datasets (Figure S9D, Table S10).

In recent 8CLC research, DPPA3, TPRX1, and ZNF280A have been linked to key regulatory roles relevant to generating 8CLCs.^36^^,^^37^ Most importantly, Mazid et al. (2022)^36^ find DPPA3 necessary for the naive to 8CLC transition. TPRX1 and ZNF280A are identified as markers of 8CLC state by both Mazid et al. (2022)^36^ and Taubenschmid-Stowers et al. (2022).^37^ We find that the LEUTX induction leads to the upregulation of DPPA3 and ZNF280A in more than one experiment produced for this paper (Figures 4B and 4C). To address the validity of the STRT-Seq data, we confirmed the upregulation of *DPPA3*, *DNMT3L*, *ZNF280A*, *DUXB*, *SGK1*, *CRX*, *NODAL*, *GNB3* and *TPRX2*, which shares high sequence similarity with *TPRX1* from the same gene famil*y*,^19^ by RT-qPCR in an independent inducible cell line with transgenic *LEUTX* (Figures 4B, S9E).

Furthermore, comparison of the recent 8CLC datasets together with our LEUTX cell models shows several potentially relevant genes. For example, CA2, CLK1, SGK1 are listed as 8CLC markers.^37^ VMO1 is undetectable in primed PSCs and 4CL naive PSCs and upregulated to moderate expression in 8CLCs in data in dataset by Mazid et al. (2022)^36^ (Figure 4C). The function of these genes in the human preimplantation development is unknown. Analysis of the embryonic ATAC-Seq data^43^supports that their expression peaks at 8-cell stage, similarly to the proposed markers DPPA3 and ZNF280A (Figure 4D).

Since we detected three key components of cohesin complex to interact with LEUTX and cohesin is bound at topologically associating domain (TAD), we cross examined our data with CCCTC-Binding factor (CTCF) binding site data and embryonic ATAC-Seq data from Wu et al. (2018).^44^ We found that LEUTX binds two sites proximal to *CRX* that coincide with CTCF binding sites. Few of the CTCF binding sites overlap LEUTX NET-CAGE enhancer peaks, indicating these binding sites were also found active in the LEUTX NET-CAGE dataset (Figure S9A). *TPRX2* is found downstream on the same strand as *CRX*, while *TPRX1* is upstream of *CRX* on the opposite strand (Figure S9A). LEUTX is bound in regions that peak in activity in the 8-cell stage, for example proximal to *TPRX2*, annotated as the *TPRX2P* pseudogene. We confirmed by RT-qPCR that LEUTX induction leads to significant *TPRX2* expression (Figure 4B).

While TPRX1 was proposed by both Mazid et al. (2022)^36^ and Taubenschmid-Stowers et al. (2022)^37^ as a key marker of 8CLC expression, neither paper discussed TPRX2 which we have found to be a upregulation target of LEUTX (Figure 4B). TPRX2 is commonly thought to be a pseudogene, but has been shown to produce mRNA product during preimplantation.^1^ Recently, Zou et al. (2022)^15^ found that combined knockdown of TPRX genes *TPRXL*, *TPRX1*, and *TPRX2* leads to delay in development and defects in EGA.

## Discussion

*LEUTX* is a primate specific gene, and one of the first genes expressed in human preimplantation embryos, its expression being restricted to the 4-cell to 8-cell stage of the preimplantation embryo.^1^^,^^17^ Of interest, in our previous studies, LEUTX appeared to be the strongest transcriptional activator among the transcription factors belonging to the same PRDL family.^17^^,^^53^ In this study, we set out to thoroughly characterize the functions of LEUTX using proteomics, transcriptomics and genomics approaches.

Unstable protein-protein interactions are difficult to capture, either because of being rare or transient in nature, or not strong enough to withstand cell lysis and affinity purification.^54^ However, through proximity labeling we could detect multiple possible chromatin-modifying complexes that are in very close contact with LEUTX. The identification of stable interactions with EP300 and CBP, together with a notable number of dynamic chromatin modifying complex interactions, provided strong evidence that LEUTX is involved in transcriptional regulation through chromatin modification, in particular histone acetylation. ChIP-Seq further confirmed that LEUTX binds close to known EP300 binding sites.

We hypothesized that LEUTX interaction with the histone acetyltransferases EP300 and CBP is mediated by the c-terminal 9aaTAD of LEUTX which is directly interacting with KIX-domains. EP300 and CBP together with MED15 are the most well-known coactivators having KIX-domains, highly conserved globular domains with three α-helices.^26^^,^^27^ KIX-domains have been found in various proteins involved in transcriptional assembly, regulation and coactivation. Currently, in UniProtKB protein database, 41 human proteins are listed as having a 9aaTAD, including embryonic transcription factors SOX9, KLF3 and ELF3 as well as all Yamanaka factors and tumor protein p53.^55^ Furthermore, p53 has previously been shown to stably interact with CBP and EP300, which is critical for its transcriptional activation potential.^56^^,^^57^ Other transcription factors with 9aaTADs and established interaction with EP300 included STAT1, STAT2^58^ and FOXO3a^59^ In this study, the removal of 9aaTAD of LEUTX eliminated the interactions with the EP300 and CBP thus confirming our hypothesis that the 9aaTAD is responsible for the direct interaction with these kinase-inducible (KIX) domains containing proteins. In addition to KIX domain, CBP has two TAZ domains and an NCBD domain that also bind 9aaTADs.^56^

Using extensive genome-wide sequencing approaches, we found that LEUTX binding sites and differentially expressed regulatory regions overlapped with a large number of repetitive elements. We found that a large number of Alu, MaLR (ERV3), and MIR (L2-end) elements overlapped LEUTX binding sites. Alu elements have previously been shown to be enriched upstream of developmental factors.^1^^,^^60^ Further, new research surrounding Alu elements shows that Alu elements are often enriched in topologically associating domain (TAD) boundaries.^61^ We detected three key members of the cohesin complex through BioID-MS and found proximity of binding sites of cohesin complex members SMC and RAD21 (ENCODE TF ChIP-Seq datasets) to LEUTX binding sites. The cohesin complex is bound at TAD boundaries, maintaining boundary formation.^62^ LEUTX was detected to interact with PRC1 complex, which together with the cohesin complex have been suggested to form TAD-like chromatin conformations, but at a smaller scale called the Polycomb-repressed domains (PRD).^63^^,^^64^ These PRDs form between Polycomb binding regions to repress transcription.^63^^,^^64^ We examined the CRX genomic locus that contains TPRX1 and TPRX2 and as such is linked to 8-cell like expression. Cross-examination of CTCF binding sites and LEUTX binding sites in this locus shows that LEUTX is bound in two CTCF binding site regions. LEUTX-induced NET-CAGE Enhancers are also overlapping with these CTCF binding sites. Many of these binding sites or enhancer regions are active in the 8-cell stage in the embryonic ATAC-Seq dataset.^44^ These findings suggest that LEUTX is possibly binding at chromatin loop boundaries which warrants further studies.

*LEUTX* and many other members of the PRD-LIKE homeobox gene family, including *ARGFX*, *DPRX*, *TPRX1* and *TPRX2* are all evolutionarily descended from the *CRX* gene.^65^ The *CRX* gene is flanked by *TPRX1* and *TPRX2* on chromosome 19, while *LEUTX* and *DPRX* have been transposed to a different location on the same chromosome, and *ARGFX* has been transposed to a different chromosome.^19^ Previous research has suggested close co-regulation or counter-regulation within the PRD-LIKE family.^17^^,^^19^^,^^53^ Maeso et al.^19^ found that human LEUTX, TPRX1 and ARGFX coregulated an largely overlapping set of genes, and Royall et al.^65^ found mCrx and mObox genes similarly coregulated overlapping set of genes, suggesting an evolved system controlling preimplantation development through the same binding site with high redundancy in at least placental mammals. In the analyses of human cells, overlapping expression and regulation profiles have been found between ARGFX, LEUTX, TPRX1 and DPRX, suggesting a role for LEUTX as a pulse-control activator, later repressed by DPRX.^17^^,^^19^^,^^53^ We also found that LEUTX upregulated its ancestral parent *CRX* and downregulated its ancestral family member *OTX2*. These all three share the same canonical DNA binding site, together with *SIX6 –* another LEUTX downregulation target. We found that the *CRX* genomic locus, also containing *TPRX1* and *TPRX2*, was under close regulation of LEUTX. Of interest, *GSC*, *CRX*, and *PITX1* become upregulated at the 8-cell stage of human development.^8^ All three share the same canonical binding site with LEUTX and follow it in temporal progression during preimplantation development. This binding site and the multitude of factors that bind it might be of key interest for preimplantation development.

We further focused our analyses on all known conserved consensus sequences for repetitive elements. In the Dfam database, 2148 repetitive element curated consensus sequences (31% out of 6915) contain the ‘GGATTA/TAATCC’ binding site. Out of the 1585 repetitive elements unique for Eutherian mammals in the Dfam database, 522 (33%) contain the ‘GGATTA/TAATCC” binding site in their consensus sequence. In the 33 repetitive elements unique to Hominidae, 25 (76%) contain the ‘GGATTA/TAATCC’ binding site. Most of the elements unique to Hominidae are AluY subtype Alu elements and ERV1 or composite retroelements. Our data suggest that the PRD-LIKE factors have possibly adopted this repetitive element binding site during Eutherian evolutionary history and are co-acting with other especially Alu element binding TFs.

Comparison of our LEUTX data to recent 8CLC sequencing data places LEUTX in a key position in understanding the molecular events of hPSCs conversion back to 8-cell stage. TPRX1, DPPA3 and ZNF280A have been indicated as key markers of 8CLCs.^36^^,^^37^ LEUTX induction leads to upregulation of DPPA3, ZNF280A and TPRX2. Overall, we found that our combined datasets support a role for LEUTX in transcriptional upregulation of 8-cell like markers and likely contributes to the transcriptional landscape of the 8-cell embryo.

In summary, we suggest that LEUTX induction causes broad downstream effects through its function as a facilitator of chromatin modification as a long-range activator binding key enhancers. LEUTX genomic binding sites overlap with regulatory regions (promoter and enhancers) and repetitive elements (Alus, MaLRs). We further show that LEUTX preferentially binds enhancer sequences, and based on protein-protein interactions, LEUTX together with CBP and EP300 likely facilitates histone acetylation. LEUTX induction leads to differential expression of several developmental transcription factors, 8-cell like markers and epigenetic modifiers that together take part in downstream embryonic development events. Our data provide an excellent resource for the LEUTX functions in human cells, as well as for researchers working with genes belonging to the same family or preimplantation development.

### Limitations of the study

We note that there are few limitations to our study. It is not possible to do functional studies that require a high number of cells in human embryos; therefore, we used several different cell lines during data collection for this study. We acknowledge that none of the cell lines exactly capture the state of the cleavage stage embryo.

To produce stable cell lines for affinity purification mass spectrometry we used the HEK293 Flp-In T-Rex cell line. This cell line allows for stable cell line production of the needed millions of cells. STRT sequencing was done in H9 cell line with the use of dCas9-activation and the biological promoter and enhancers identified for DUX4.^9^ Even with this activation method more closely mimicking biological expression, culture system does not mimic the actual context of cleavage stage embryo. The same factors and genomic regions may not be active as in the cleavage stage embryo. NET-CAGE and ChIP-Seq were done in hIPSC line HEL24.3. Similarly, transcriptional conditions are not the same in this cell line and cleavage stage embryo. Although combination of different model systems allows us to capture the conserved features that are independent of cell line, both H9 and HEL24.3 are imperfect models of the cleavage stage embryo.

The questions whether LEUTX is an essential transcription factor in early human development, whether LEUTX is necessary for the pluripotent-to-totipotent transition or whether it induces a distinct early-embryonic-like state in hPSC remain to be resolved. In addition, our study had technological limitations. It is currently not feasible to perform NET-CAGE or mass spectrometry-based interactome analyses in 8CLC cell models in which only small number of cells are converted to 8-cell like cells. The methods require a large number of cells for the library preparation or data collection.^23^ Therefore, further studies are needed to further model the function of LEUTX in human preimplantation development.

The experiments detailed in this paper cannot address the exact molecular function of LEUTX during the 4- and 8-cell stages, nor can it address how LEUTX affects its transcriptional regulation. How LEUTX regulates transcription on a biochemical level, *in vivo* function of LEUTX and LEUTX function in 8CLC merits further study.

## STAR★Methods

### Key resources table

**Table:** 

REAGENT or RESOURCE	SOURCE	IDENTIFIER
**Antibodies**		

anti-HA.11 epitope tag antibody	Biolegend	# 901502; RRID:AB_2565007
mouse IgG	Santa Cruz	# sc-2025; RRID:AB_737182

**Chemicals, peptides, and recombinant proteins**		

FuGENE® 6 transfection reagent	Promega	Cat# E2691
Hygromycin B	Invitrogen	Cat# 10687-010
Tetracycline hydrochloride	Sigma-Aldrich	Cat# T-3383
Biotin	Pierce	Cat# 29129
Protease Inhibitor cocktail	Sigma-Aldrich	Cat# P8340
Benzonase Nuclease	Santa Cruz	Cat# sc-202391
Sequencing grade modified porcine trypsin	Promega	Cat# V5113
N-dodecyl-β-d-maltoside	Sigma-Aldrich	Cat# D4641-5G
HEPES buffer pH8.0	ITW	Cat# A69060250
Sodium fluoride (NaF)	Sigma-Aldrich	Cat# S7920-500G
Ethylenediaminetetraacetic acid disodium salt (EDTA)	Chemsupply	Cat# 9326410003617
Phenylmethanesulfonyl fluoride (PMSF)	Sigma-Aldrich	Cat# P7626
Trifluoroacetic acid (TFA); sequencing grade, 10 × 1 ml	Thermo Fisher Scientific	Cat# PIE28904
Acetonitrile, Optima LC/MS-grade	Thermo Fisher Scientific	Cat# FSBA955-4
Geltrex LDEV-Free, hESC-Qualified, Reduced Growth Factor Basement Membrane Matrix	Thermo Fisher Scientific	Cat# A1413302
Essential 8 Medium	Thermo Fisher Scientific	Cat# A1517001
Doxycycline hyclate	Sigma Aldrich	Cat# D9891
UltraPure 0.5M EDTA, Ph 8.0	Thermo Fisher Scientific	Cat# 15575020
Trimethoprim	Sigma-Aldrich	Cat# T7883
TrypLE Express Enzyme	Thermo Fisher Scientific	Cat# 12604-021
Pierce™ 16% Formaldehyde (w/v), Methanol-free	Thermo Fisher Scientific	Cat# 28906
Penicillin–streptomycin	Life Technologies	Cat# 5140130

**Critical commercial assays**		

Gateway BP Clonase Enzyme Mix	Life Technologies	Cat# 11789021
Gateway LR Clonase Enzyme Mix	Life Technologies	Cat# 11791043
Strep-Tactin Sepharose 50% (vol/vol) suspension	IBA Life Sciences	Cat# 2-1201-010
NucleoSpin Plasmid EasyPure	Macheney-Nagel	Cat# 740727.250
Neon transfection system 100 μl kit	Thermo Fisher Scientific	Cat# MPK10096
Nextera DNA sample preparation kit, Illumina	Illumina	Cat# FC-121-1030
Nextera DNA Library Prep	Illumina	Cat# 15028212
NextSeq 500/550 High Output kit v2.5 (75 cycles)	Illumina	Cat# 20024906
NucleoSpin Gel and PCR purification Kit	Macheney-Nagel	Cat# 740609
NucleoSpin RNA Plus	Macheney-Nagel	Cat# 740984
HOT FIREpol qPCR Master Mix	Solis Biodyne	Cat# 08-25-00020
GeneJET Plasmid Miniprep Kit	Thermo Fisher Scientific	Cat# K0503

**Deposited data**		

LEUTX STRT-Sequencing data	This paper	E-MTAB-10539
LEUTX NET-CAGE-Sequencing data	This paper	PRJEB45266
LEUTX ChIP-Sequencing data	This paper	PRJEB45266
LEUTX; LEUTX-K57A; LEUTX-9aaTAD proteomics data	This paper	MSV000087381
Enhancer annotation: dbSuper	Khan and Zhang, 2016^43^	https://asntech.org/dbsuper/
FANTOM5 Promoter CAGE Peaks and Human permissive enhancers phase 1 and 2	Lizio et al., 2015^42^	https://fantom.gsc.riken.jp/5/
Embryonic ATAC-Seq data	Wu et al., 2018^44^	GSE101571
ChIP-Seq Integrative Genomics Viewer datasets	Robinson et al., 2011^66^	Gm06990 (CTCF, HUVEC CTCF, and K562 CTCF)
ENCODE ChIP-Seq TF dataset	https://www.encodeproject.org	ENCFF563SWF (ARID3A_K562)
ENCODE ChIP-Seq TF dataset	https://www.encodeproject.org	ENCFF879ZMI (ARID2_K562)
ENCODE ChIP-Seq TF dataset	https://www.encodeproject.org	ENCFF113BTA (YY1_H1)
ENCODE ChIP-Seq TF dataset	https://www.encodeproject.org	ENCFF792HJJ (NFRKB_HEK293T)
ENCODE ChIP-Seq TF dataset	https://www.encodeproject.org	ENCFF786IZD (ZNF462_GM23338)
ENCODE ChIP-Seq TF dataset	https://www.encodeproject.org	ENCFF970MYF (KLF5_GM12878)
ENCODE ChIP-Seq TF dataset	https://www.encodeproject.org	ENCFF914NEO (SP2_H1)
ENCODE ChIP-Seq TF dataset	https://www.encodeproject.org	ENCFF305PPC (SP1_H1)
ENCODE ChIP-Seq TF dataset	https://www.encodeproject.org	ENCFF532VPN (CREBBP_K562)
ENCODE ChIP-Seq TF dataset	https://www.encodeproject.org	ENCFF539ZQW (EP300_K562)
ENCODE ChIP-Seq TF dataset	https://www.encodeproject.org	ENCFF726NGV (EP300_HepG2)
ENCODE ChIP-Seq TF dataset	https://www.encodeproject.org	ENCFF307PSW (EP300_HepG2)
ENCODE ChIP-Seq TF dataset	https://www.encodeproject.org	ENCFF899RKF (EP300_K562)
ENCODE ChIP-Seq TF dataset	https://www.encodeproject.org	ENCFF840MWN (EP300_H1)
ENCODE ChIP-Seq TF dataset	https://www.encodeproject.org	ENCFF492IMA (SMC3_HepG2)
ENCODE ChIP-Seq TF dataset	https://www.encodeproject.org	ENCFF289LLT (SMC3_K562)
ENCODE ChIP-Seq TF dataset	https://www.encodeproject.org	ENCFF532ZYE (RAD21_H1)
ENCODE ChIP-Seq TF dataset	https://www.encodeproject.org	ENCFF960TEU (RAD21_K562).

**Experimental models: Cell lines**		

Human: HEK Flip-In T-REx 293	Invitrogen, Life Technologies	R78007
Human: HEK-293	ATCC	Cat# CRL-1573
Human: LEUTX-TetON human ES cell: WA09	This paper	N/A
Human: HA/V5 tagged LEUTX-TetON human iPSC: HEL24.3	This paper	N/A

**Recombinant DNA**		

pB-tetON-bgi-LEUTX-ires-GFP-PGK-Puro	This paper	N/A
pB-tetON-bgi-LEUTXw/o9aaTAD-ires-GFP-PGK-Puro	This paper	N/A
pB-tetON-bgi-LEUTX-V5-HA-IRES-GFP-PGK-Puro	This paper	N/A
SB-tight-DDdCas9VP192- GFP-Zeo-WPRE	This paper	N/A
SB-CAG-rtTA-IN-IRES-Neo	This paper	N/A
CAG-SB-100X-bghpA	This paper	N/A
pCMV-HAhy-Pbase	This paper	N/A
GGdest	Addgene Balboa et al.,2015^67^	Cat# 69538
LEUTX pDONR 221	This paper	N/A
LEUTX-9aaTADdel pDONR 221	This paper	N/A
LEUTX-K57A pDONR 221	This paper	N/A
LEUTX-MAC-C	This paper	N/A
LEUTX-9aaTADdel-MAC-C	This paper	N/A
LEUTX-K57A-MAC-C	This paper	N/A
GFP-NLS-MAC-C	This paper	N/A
MAC-tag-C destination vector	Addgene	108077
Gateway pDONR221	Thermo Fisher Scientific	Cat# 12536017
pOG44 Flp-Recombinase Expression Vector	Life Technologies	Cat# V600520

**Software and algorithms**		

Picard v2.20.4	https://github.com/broadinstitute/picard	http://broadinstitute.github.io/picard/
HISAT2 v2.1.0	Kim et al., 2019^68^	https://daehwankimlab.github.io/hisat2/
SAMtools v1.9	Li et al., 2009^69^	http://www.htslib.org/
BEDtools v2.27.1	Quinlan and Hall. 2010^70^	http://bedtools.readthedocs.io/
featureCounts v1.5.2	Liao et al., 2014.^71^	http://subread.sourceforge.net/
StringTie v1.3.3	Pertea et al., 2015^72^	https://ccb.jhu.edu/software/stringtie/
R v4.0.1	R core Team 2020^73^	https://www.r-project.org/
edgeR v3.30.3	Robinson et al., 2010^74^	http://bioconductor.org/packages/release/bioc/html/edgeR.html
ggplot2 v3.3.2	Wickham et al. 2016.^75^	https://ggplot2.tidyverse.org/
ChIPseeker v.1.24.0	Yu et al. 2015^39^	https://www.bioconductor.org/packages/release/bioc/html/ChIPseeker.html
Pymol v.2.3	Schrödringer LCC	https://pymol.org
Coot v. 0.8.9.2	Emsley et al., 2010^76^	https://www2.mrc-lmb.cam.ac.uk/personal/pemsley/coot/
RUVSeq v.1.22.0	Risso et al. 2014^77^	https://www.bioconductor.org/packages/release/bioc/html/RUVSeq.html
EnhancedVolcano v.1.7.16	Blighe et al. 2020^78^	https://www.bioconductor.org/packages/release/bioc/html/EnhancedVolcano.html
Gviz v.1.32.0	Hahne and Ivanek. 2016^79^	https://www.bioconductor.org/packages/release/bioc/html/Gviz.html
enrichR v3.0	Xie et al. 2021^80^	https://cran.r-project.org/web/packages/enrichR/index.html
rrvgo v.1.0.2	Sayols 2020^81^	https://bioconductor.org/packages/release/bioc/html/rrvgo.html
SAINTexpress v. 3.6.3	Teo et al. 2013^82^	http://saint-apms.sourceforge.net/Main.html
XCalibur v. 3.0.63	Thermo Fisher Scientific	https://www.thermofisher.com/order/catalog/product/OPTON-30965#/OPTON-30965
Proteome Discoverer v.1.4	Thermo Fisher Scientific	https://www.thermofisher.com/fi/en/home/industrial/mass-spectrometry/liquid-chromatography-mass-spectrometry-lc-ms/lc-ms-software/multi-omics-data-analysis/proteome-discoverer-software.html
Cytoscape v3.6.	Shannon et al. 2003^83^	https://cytoscape.org/
HOMER v4.11	Heinz et al. 2010^84^	http://homer.ucsd.edu/homer/
DeepTools v.3.5	Ramírez et al. 2016^85^	https://deeptools.readthedocs.io/en/develop/
MACS2 v.2.2.7.1	Zhang et al. 2008^86^	https://pypi.org/project/MACS2/
Bowtie2 v.2.4.1	Langmead et al. 2012^87^	http://bowtie-bio.sourceforge.net/bowtie2/index.shtml
STAR v 2.5.0a	Dobin et al. 2013^88^	https://github.com/alexdobin/STAR
Cutadapt v 1.1.8	Martin 2011	http://code.google.com/p/cutadapt/

**Other**		

Bio-Spin Chromatography Columns	Bio-Rad	Cat# 732-6008
100-mm-long reversed-phase C18 end-capped HPLC column	Merck	Cat# 1021290001
Autosampler vials for MS	Thermo Fisher Scientific	Cat# THC160134

### Resource availability

#### Lead contact

Further information and requests for resources and reagents should be directed to and will be fulfilled by the lead contact, Juha Kere (juha.kere@ki.se).

#### Materials availability

Plasmids generated in the study will be available upon request.

### Experimental model and subject details

#### Cell lines

##### HEK Flip-In T-REx 293

Stable cell line used in proteomics experiments, Male (Invitrogen, Life Technologies, R78007). Cultured in 37°C, low glucose DMEM, with 1% Streptomycin and 10% FSB.

##### H9

hESC female cell line (WA09, WiCell), used in STRT experiments. Cells were maintained on Geltrex, hESC-qualified, reduced growth factor basement membrane matrix-coated tissue culture dishes in Essential 8 culture medium and passaged every three to five days by 3-5-min incubation with 0.5 mM EDTA (all from Thermo Fisher Scientific). Cultured in 37°C, 5% CO_2_ in a humidified atmosphere.

##### HEL24.3

Locally produced hiPSC line HEL24.3,^89^ Male, used in the NET-CAGE and ChIP-Seq experiments, was maintained on Geltrex, hESC-qualified, reduced growth factor basement membrane matrix-coated tissue culture dishes in Essential 8 culture medium and passaged every three to five days by 3-5-min incubation with 0.5 mM EDTA (all from Thermo Fisher Scientific). Cultured in 37°C, 5% CO_2_ in a humidified atmosphere.

##### Human samples

No human samples were used in this study.

##### Animal models

No animal experiments were performed in this study.

### Method details

#### Cloning of vectors for LEUTX overexpression

In order to overexpress *LEUTX* in human pluripotent cells, the ORF was cloned into a modified piggyBac vector. *LEUTX* ORF was amplified from a TOPO vector containing the full-length clone (European nucleotide archive accession numbers: LN651090). The PCR product was ligated into piggyBac vector. The final vector was called pB-tetON-bgi-LEUTX-ires-GFP-PGK-Puro.LEUTX. The ORF was further modified by removing the C-teminal 9 amino acid TAD. The ORF was amplified form a TOPO vector containing full length clone LN651090. The PCR product was digested using AgeI and NotI and ligated into piggyBac vector. The final vector was called pB-tetON-bgi-LEUTXw/o9aaTAD-ires-GFP-PGK-Puro. For ChIP-seq, C-terminal V5 and HA tags were added to wild type *LEUTX*. The ORF was amplified in two-step PCR using pB-tetON-bgi-LEUTX-ires-GFP-PGK-Puro as a template. The PCR product was digested using AgeI and NotI and ligated into piggyBac vector. The final vector was called pB-tetON-bgi-LEUTX-V5-HA-IRES-GFP-PGK-Puro. Primers reported in Table S12.

#### Cloning of LEUTX to MAC-tag Gateway® destination vector for mass spectrometry

The wild type LEUTX and mutants were first amplified in a two-step PCR reaction from vectors above and cloned into a Gateway compatible entry clone using Gateway BP Clonase II (Invitrogen) according to manufacturer’s instructions (Primers in Table S12). The entry clone was further cloned to Gateway compatible destination vector containing the C-terminal MAC-tag (Addgene #108077).^20^^,^^29^

#### Cell culture for mass spectrometry

To produce stable cell lines stably expressing MAC-tagged LEUTX, Flip-In T-REx 293 cell lines (Invitrogen, Life Technologies, R78007, cultured in manufacturer’s recommended conditions) were co-transfected with the expression vector and the pOG44 vector (Invitrogen) using Fugene6 transfection reagent (Roche Applied Science). One day after transfection, cells were selected in 1% Streptomycin and 100 μg/ml Hygromycin for two weeks after which positive clones were pooled and amplified. Green fluorescent protein (GFP) tagged with MAC-tag was used as a negative control and processed parallel to the bait proteins. Stable cell line was expanded to 80% confluence in 20 × 150mm cell culture plates. Ten plates were used for AP-MS, in which 2 μg/ml tetracycline was added for 24 h induction, and ten plates for BioID, in which 50 μM biotin in addition to tetracycline, was added for 24 h before harvesting. Cells from five fully confluent dishes were pelleted as one biological sample. In total two biological replicates in two different approaches were produced. Samples were snap frozen and stored at –80°C.

#### Affinity purification mass spectrometry

In the AP-MS sample purification the sample was lysed in 3 ml ice-cold Lysis Buffer I (1% n-Dodecyl beta-D-maltoside, 50mM Hepes, pH 8.0, 150 mM NaCl, 50 mM NaF, 1.5 mM NaVo_3_, 5 mM EDTA, 0.5 mM PMSF and Sigma Proteinase Inhibitor). In the BioID-MS sample the cell sample was lysed in 3 ml ice-cold Lysis Buffer I, supplemented with 1 μl Benzonase per sample and sonicated in a water bath in cycles with 3x continuous sonication and 5min break. Lysed samples were centrifuged at 16000x for 15 min, and again 10 min to produce cleared lysate, that was loaded on Bio-Rad spin columns that had 400 μl Strep-Tactinbeads (IBA, GmbH) prewashed with Lysis Buffer I. The loaded beads were washed 3 × 1 ml with Lysis Buffer I, and 4 × 1 ml with Wash Buffer (50 mM tris-HCl, pH 8.0, 150 mM NaCl, 50 mM NaF, 5 mM EDTA). To eluate sample, the beads were resuspended in 2 × 300 μl Elution Buffer (50 mM Tris-HCl, pH 8.0, 150 mM NaCl, 50 mM NaF, 5 mM EDTA, 0.5 mM Biotin) for 5 min and eluates were collected into an Eppendorf tube, followed by a reduction of the cysteine bonds with 5mM Tris(2-carboxyethyl)phosphine (TCEP) for 30 min at 37°C and alkylation with 10 mM iodoacetamide. The proteins were then digested to peptides with sequencing grade modified trypsin (Promega, V5113) at 37°C overnight. Samples were then desalted by C18 reversed-phase spin columns according to manufacturer’s instructions. The sample was dried in a vacuum centrifuge and reconstituted to a final volume of 30 μl in 0.1% TFA and 1% Acetonitrile. More detailed protocol can be found in Liu et al., (2020).^29^

#### Liquid chromatography-mass spectrometry (LC-MS)

Analysis was performed on a Q-Exactive mass spectrometer with an EASY-nLC 1000 Liquid Chromatograph Q Exactive™ Hybrid Quadrupole-Orbitrap™ system via an electrospray ionization sprayer (Thermo Fisher Scientific), using Xcalibur version 3.0.63 as described in Liu et al. (2018).^20^ Database search was performed with Proteome Discoverer 1.4 (Thermo Scientific) using the SEQUEST search engine on the Reviewed human proteome in UniProtKB/SwissProt databases (http://www.uniprot.org, downloaded Nov. 2020). Trypsin was selected as the cleavage enzyme and maximum of 2 missed cleavages were permitted, precursor mass tolerance at ±15 ppm and fragment mass tolerance at 0.05 Da. Carbamidomethylation of cysteine was defined as a static modification. Oxidation of methionine and for BioID samples biotinylation of lysine and N-termini were set as variable modifications. All reported data were based on high-confidence peptides assigned in MSFragger v17 (FDR < 0.01).

#### Validation of promoters and enhancers using CRISPRa

Putative *LEUTX* enhancer regions 1 and 2 were predicted from Tet-On DUX4 hESC NET-CAGE dataset.^9^ Putative *CRX* enhancer and promoter regions were predicted from NET-CAGE data introduced in this study. The guide RNAs targeting the each of the putative enhancers or promoters were designed using the Benchling CRISPR tool (https://benchling.com), targeting them to the proximal promoters (−400 to −50 base pairs from transcription start site) or +/−200 base pairs of the putative enhancer midpoint. Guide sequences were selected according to their on- and off-target score and position. Guide RNA transcriptional units (gRNA-PCR) were prepared by PCR amplification with Phusion polymerase (Thermo Fisher), using as template U6 promoter and terminator PCR products amplified from pX335 together with a guide RNA sequence-containing oligo to bridge the gap. The oligos for guide RNA transcriptional units are as in (Balboa et al., 2015).^67^ PCR reaction contained 50 pmol forward and reverse primers, 2 pmol guide oligo, 5 ng U6 promoter and 5 ng terminator PCR products in a total reaction volume of 100μL. The PCR reaction program was 98°C/10 sec, 56°C/30 sec, 72°C/12 sec for 35 cycles. Amplified gRNA-PCRs were purified and transfected to HEK293 cells.

HEK 293 cells were seeded on tissue culture treated 24-well plates one day prior to transfection (5 × 104 cells/well). Cells were transfected using FuGENE HD transfection reagent (Promega) in fibroblast culture medium with 500 ng of dCas9VP192 transactivator encoding plasmid and 200 ng of guide RNA-PCR product or TdTomato guide RNA plasmid. Cells were cultured for 72 h post-transfection, after which samples were collected for qRT-PCR. Successful activation of *LEUTX* and *CRX* was confirmed by qPCR.

In order to introduce *LEUTX* guides to DD-dCas9 activator cell line, guide cassettes containing either four guide oligos targeting *LEUTX* promoter or five guide oligos targeting enhancers 1 or 2 were assembled in a GoldenGate reaction using the four different *LEUTX* promoter guide oligos and 5 different guide oligos targeting enhancers 1 and 2 as described in (Balboa et al., 2015).^67^ Guide cassettes containing both promoter and enhancer guides was further cloned together. Finally, the guide cassettes were cloned to piggyBac vector. Primer sequences for promoter and enhancer guide oligos are provided in Table S12. See Figure S5 for LEUTX enhancer validation.

#### Generation of TetOn LEUTX hPSCs

Inducible LEUTX cell lines used for NET-CAGE and ChIP-Seq were generated on hiPSC line HEL24.3. Inducible dCas9-activator cell line for endogenous gene activation was generated on hESC line H9 (WA09, WiCell).

HEL24.3. and H9 cells were treated with 10 μM ROCK inhibitor Y27632 (Selleckhem) for 4 h before electroporations. Cells were incubated with StemPro Accutase (Thermo Fisher Scientific) until the edges of the colonies started to curl up. The Accutase was aspirated and the cells were gently detached in cold 5% FBS (Thermo Fisher Scientific) 1×PBS (Corning) and counted. One million cells were centrifuged at 200xg for 5 min and the pellet was transferred into 120 μl of R-buffer containing 1 μg of either one of the LEUTX vectors (pB-tetON-LEUTX-ires-GFP-PGK-Puro/ pB-tetON-LEUTX-HA-V5-ires-GFP-PGK-Puro) or DDdCas9 plasmid cocktail below and 0.5 μg of transposase plasmid. 100 μl of the cell-plasmid suspension was electroporated with two pulses of 1100V, 20 ms pulse width, using Neon Transfection system (Thermo Fischer Scientific). Activator cell line was generated by electroporating H9 cells with two plasmids containing DDdCas9VP192 (1 μg of SB-tight-DDdCas9VP192- GFP-Zeo-WPRE) and rtTA (1 μg of SB-CAG-rtTA-IN-IRES-Neo) sequences, which were integrated into the genome by sleeping beauty transposase (0.5 μg of CAG-SB-100X-bghpA). Guide plasmids (1.5 μg / reaction) were electroporated into H9 DDdCas9VP192 activator cells and integrated with piggyBac transposase (0.5 μg of pCMV-HAhy-Pbase).

The electroporated cells were plated on Geltrex-coated dishes in Essential 8 medium with 10 μM ROCK inhibitor Y27632. The following day, the medium was exchanged with fresh Essential 8 medium without ROCK inhibitor. The cells were selected with Neomycin (G418, Life Technologies) at 50 μg/ml and Zeocin (Sigma) at 1 μg/ml (after DDdCas9VP192-GFP-Zeo-WPRE plasmid transfection) or Puromycin (Sigma) at 0.5 μg/ml (after LEUTX vectors and guide plasmids). The TetOn-LEUTX hPSC clones were picked manually on Geltrex-coated 24-well plates, expanded and selected again with Puromycin. Appearance of the GFP reporter protein was tested using Doxicycline at concentration 0.5 μg/ml and detected using an EVOS FL Cell imaging system (Thermo Fisher Scientific).

For the experiments presented in this paper, the LEUTX TetOn cells were treated with 1 μg/ml of Doxycycline for 6-7 h (NET-CAGE, q-PCR validation) or 24 h (ChIP-Seq, qPCR validation), DD-dCas9 activator cell line was treated with 1 μg/ml of Doxycycline and 1 μM Trimethoprim for 24 h, 48 h or 72 h, prior to harvesting cells for STRT-Seq.

#### NET-CAGE library preparation and sequencing

Nascent RNA from flash-frozen cells was isolated as described by Hirabayashi et al. (2019)^23^ with the following exceptions: (i) 5× DNase I enzyme (Thermo Fisher Scientific) was used to prepare the DNase I solution (50 μl), (ii) the samples were incubated for up to 1hat 37°C while being pipetted up and down several times every 10 min, and (iii) RNA quality was measured using TapeStation 4200 (Agilent). CAGE-based libraries were generated according to the no-amplification non-tagging CAGE libraries for Illumina next-generation sequencers (nAnT-iCAGE) protocol. All CAGE-based libraries were sequenced in single-read mode on an Illumina NextSeq500 platform.

#### ChIP-seq cell culture and chromatin shearing

HEL24.3 TetOn LEUTX cells were expanded on Geltrex coated tissue culture dishes in Essential 8 culture medium and treated with 1 μg/ml of Doxycycline for 24 h prior to fixation. Cells were detached from four confluent 10 cm plates with and without doxycycline treatment using TrypLE (Thermo Fisher Scientific). ChIP assays were performed as previously described.^24^ Cells were fixed in 1% formaldehyde (Thermo Fisher Scientific) for 10minat room temperature and washed twice with ice-cold PBS. The cell pellet was resuspended for lysis in RIPA buffer. Cross-linked chromatin was sonicated to an average fragment size of 200-500 bp then was immunoprecipitated with anti-HA.11 epitope tag antibody (Biolegend, # 901502) and mouse IgG antibody (Santa Cruz, # sc-2025) in LEUTX-V5-HA overexpressed (Dox+) and non-treated (Dox-) iPSCs respectively. ChIP libraries were prepared according to Illumina’s instructions and were sequenced using Illumina NextSeq 500 at Biomedicum Functional Genomics Unit (FuGU).

#### Modified STRT RNA-seq library preparation and sequencing

For the RNA-seq we used a modified version^90^ of a previously described single-cell tagged reverse transcription (STRT) protocol with unique molecular identifiers (UMIs).^21^^,^^22^ Briefly, we used 20 ng of RNA to generate a 48-plex barcoded RNA-seq library: we placed the RNA samples on a 48-well plate and added a universal primer, template-switching oligonucleotides, and a 6-bp barcode sequence (for sample identification) to each well of the plate.^91^ We pooled the synthesized cDNAs into one library, performed fragmentation to 200–400 bp (Covaris), captured the 5′-prime fragments, added an adapter, and amplified the targets by PCR. The RNA-seq library was sequenced with Illumina NextSeq 500 System, High Output (75 cycles) and the service was provided by the Biomedicum Functional Genomics Unit at the Helsinki Institute of Life Science and Biocenter Finland at the University of Helsinki.

#### Quantitative RT-PCR (qPCR)

For real-time SYBR-Green based qPCR total RNA was extracted using NucleoSpin RNA Plus kit (Macherey-Nagel). Total RNA was reverse-transcribed into cDNA by M-MLV Reverse Transcriptase (Promega) in RT reaction containing Random hexamers (Promega), Oligo (dT) 18 Primer (Thermo Scientific), the mix of all 4 dNTPs and Riboblock RNAse inhibitor (Thermo Scientific). The cDNA amount was determined as the synthesized cDNA in a 20 μl RT-reaction containing 1 μg total RNA.

Gene expression was assessed using SYBR-Green based qRT-PCR. The reactions for the qPCR were prepared with a Corbett CAS-1200 liquid handling system and the qPCR was performed using Corbett Rotor-Gene 6000 (Corbett Life Science, Sydney, Australia) with a thermal cycle of 95°C for 15 min, followed by 40 cycles of 95°C 25 s, 60°C 25 s, 72°C 25 s, followed by a melting step. Relative quantification of gene expression was performed following the ΔΔCt method with housekeeping gene Cyclophilin G as an endogenous control. All qPCR primers are listed in Table S12.

### Quantification and statistical analysis

#### Structural modeling

The predicted interactions of K57 and K57A with the HD of LEUTX and dsDNA motif are based on the model structure reported in Katayama et al., (2018).14 The model of the LEUTX 9aaTAD peptide bound to the KIX domain of CBP is based on the NMR structure of the CBP KIX domain in complex with the MLL and pKID peptides (PDB code 2LXT, model 1/20; 28): the MLL peptide 847SDIMDFVLK855 was mutated to match the LEUTX 9aaTAD 178SSLNQYLFP186 (UniProt ID: A8MZ59) using PyMOL (version 2.3; Schrödinger LLC) and Coot (version 0.8.9.2; 70); the coordinates of the additional residues of the MLL peptide, and the entire pKID peptides were removed. PDB coordinates for KIX in complex with LEUTX 9aaTAD is available in the Table S13.

#### Proteomics: Identification of statistical confidence of interactions

Significance Analysis of INTeractome (SAINT) -express version 3.6.3 and Contaminant Repository for Affinity Purification (CRAPome, http://www.crapome.org) were used to discover statistically significant interactions from the AP-MS data. The LEUTX LC-MS data was ran together with a large GFP control set. Final results represent proteins with a SaintScore > 0.74, and in less than 20% of Crapome database experiments except in cases where AvgSpec is three times higher than AvgSpec in Crapome experiments. Protein interaction networks were constructed from filtered SAINT data that was imported to Cytoscape 3.6.0.^83^ Known prey-prey interactions were obtained from the iRef database (http://irefindex.org).

#### Proteomics: Overrepresentation analysis

Enrichment analysis were done with statistically filtered (see above) list of protein-protein interactions. GO annotation enrichment analysis for protein-protein interaction data was done with EnrichR, rrvgo R-package was used to reduce the number of GO-terms into their parent terms.^80^ CORUM^32^ enrichment for protein-protein interaction data was done using EnrichR. Plots were drawn using R-package ggplot2.^75^ Preys were compared against the EpiFactors database (https://epifactors.autosome.ru/) for known epigenetic function (downloaded 1/2021).^33^

#### NET-CAGE read-alignment for CAGE-based data

Reads were split by barcode using the MOIRAI package. Cutadapt v 1.1.8 (http://code.google.com/p/cutadapt/)^92^ was used to trim reads to 73 bp and remove reads below base quality 33 and ‘N’ bases. Reads aligning to ribosomal RNA sequences (GenBank U13369.1) were removed using the rRNAdust script within the MOIRAI package. The resulting reads were aligned to the human genome (hg19) using STAR v 2.5.0a^75^with Gencode v27lift37 (“comprehensive”) as the reference gene model. Mapping was performed with the following parameters: --runThreadN 12 --outSAMtype BAM SortedByCoordinate --out FilterMultimapNmax 1. Following alignment, the technical replicates were merged using the Picard Toolkit v 2.0.1 with the MergeSamFiles program (Broad Institute, Picard Toolkit, 2018. http://broadinstitute.github.io/picard).

#### NET-CAGE identification of transcribed promoters and enhancers

Reads mapping to known FANTOM5 promoters and FANTOM-NET enhancers were counted and 1normalized essentially as described in Hirabayashi et al., (2019).^23^ Decomposition peak identification (https://github.com/hkawaji/dpi1/blob/master/identify_tss_peaks.sh) was used to identify tag clusters with default parameters but without decomposition. Peaks with at least three supporting CAGE tags were retained and used as input to identify bidirectional enhancers (https://github.com/anderssonrobin/enhancers/blob/master/scripts/bidir_enhancers).

#### NET-CAGE statistical analysis

To found differentially expressed promoters and enhancers, we normalized to library size and kept peaks that have been detected in at least two samples and have log2CPM > -2.5 (enhancers) log2CPM > -2 (promoters). Differentially expressed peaks represent those that have FDR < 0.05 with EdgeR Generalized Linear Model Likelihood Ratio Test.^74^ Upregulated and downregulated differentially expressed promoters and enhancers were defined as logFC>0 and logFC<0 respectively.

#### STRT alignment

The sequenced raw reads were processed using the STRT2 pipeline.^90^ Briefly, base call (BCL) files were demultiplexed and converted to FASTQ files with Picard tools (v2.20.4; http://broadinstitute.github.io/picard/), and aligned to the human reference genome hg19, ribosomal DNA unit (GenBank: U13369), and ERCC spike-ins (SRM 2374) with the GENCODE (v28) transcript annotation by HISAT2 (v2.1.0).^68^ For gene-based analysis, uniquely mapped reads within the 5′-UTR or 500 bp upstream of the protein-coding genes and the first 50 bp of spike-in sequences were counted with featureCounts (v1.5.2).^71^ For TFE-based analysis, the mapped reads were assembled by StringTie (v1.3.3)^81^ and those mapped reads within the first exons of the assembled transcripts were counted as previously described in Töhönen et al.^1^ FASTQ files after exclusion of duplicated reads were deposited in the ArrayExpress database at EMBL-EBI (https://www.ebi.ac.uk/arrayexpress) under accession number E-MTAB-10539.

#### STRT differential expression analysis

Normalized to RNA Spike-ins with the R-Package RUVSeq.^77^ During initial analysis and normalization, we found that the first row of the PCR plate (first 8 samples) were notably different from the rest of the samples. To keep the sampletype amounts the same (promoter, promoter + enhancer, promoter + enhancer2) we excluded the first 12 samples from the analysis and for the TFE tables samples were realigned with first 12 samples removed (Figure S10). Filtered out very lowly expressed genes by requiring more than 5 reads in at least two samples. We used a model accounting for the RNA Spike ins, pipetting set (set/time of pipetting), and the sampletype (Promoter only, Promoter + Enhancer1, Promoter + Enhancer2). EdgeR genewise negative binomial generalized linear models with quasi-likelihood test. Differentially expressed genes and TFEs are defined as those with FDR < 0.05.

#### ChIP-seq alignment and statistical analysis

The sequence alignment was done by Bowtie 2^77^ using GRCh38 as reference human genome and the ChIP-seq peak calling was carried out using the MACS2^86^ (Figure S11). MACS2 peaks with FDR < 0.05 were considered significant. MACS2 peaks were transferred to hg19 using LiftOver to be compared with the other genomic data sets.

#### Annotation on genomic regions

Annotation plots for genomic regions were done with ChIPSeeker R-package,^39^ with promoter regions defined as 3000 kb up or downstream from known GENCODE TSS sites. Plotting of genomic regions was done using Gviz R-package^79^ and using Integrative Genomics Viewer.^66^

#### Motif finding: MEME suite

To analyze which motifs were found in the genomic coordinates we had we used MEMESuite.^93^ TFE and Promoters were extended with 2500bp up- and 500 downstream of peak coordinates, Enhancers peaks were extended 500bp up and downstream, whereas ChIP-Seq peaks were not extended. MEME^94^ for all genomic data was run with settings mode:”anr”, nmotifs = 25, min width = 6, maxwidth = 50, minimum sites 50, csites = 3000, time = 30000. Further, we analyzed what motifs were enriched in each data set with the MemeSuite tool SpaMo. SpaMo was run with default settings using the motif database HOCOMOCO core human version v11.

#### Repetitive elements overlap

Repetitive elements RepMasker track was downloaded from UCSC Genome Browser. Bedtools^70^ was used to see if any genomic locations overlap with repetitive elements (hg19). Only genomic coordinates that directly overlap with a repetitive element (distance 0) are considered “overlapping”. Categorization of Repetitive Element subtypes was done through the Categories from the Dfam database (https://www.dfam.org/, downloaded 6/2020). For this analysis the length of promoters was extended by 130 bp in each direction to bring the average length more in line with other types of data. Average peak lengths were STRT TFE: 297.6 bp, NET-CAGE enhancer 336.6 bp, NET-CAGE promoter 31.8, ChIP-Seq peaks 208.3. After extension the average NET-CAGE promoter was 291 bp. All promoters (Promoter CAGE Peaks) and enhancers (Human permissive enhancers phase 1 and 2) were downloaded from FANTOM5 (https://fantom.gsc.riken.jp/5/ , downloaded 8/2020). Promoters were extended by 130 bp in each direction, chrM promoters were excluded. Bedtools was used as mentioned above to produce overlap profiles for ‘all promoters’ and ‘all enhancers’ that were then compared pairwise with our NET-CAGE results with Chi-squared test in an inhouse R-script. HOMER Repeat annotation for each genomic data were done through HOMER annotatePeaks.pl using GENCODE hg19 gtf file (https://www.gencodegenes.org/human/, downloaded 1/2021) and the “-genomeOntology” setting.^84^ HOMER Genome Ontology search searches for enrichment of genomic annotations in searched regions, including repetitive elements.

#### Enhancer annotation: dbSuper

The Super enhancer database dbSUPER^43^ was used to see if our identified regulatory regions overlap with known super enhancers. We downloaded the BED-files for H1 and H9 datasets (h19) and used Bedtools^70^ overlap to check for overlap with distance 0 considered overlap. (https://asntech.org/dbsuper/, downloaded 8/2020). H1 dataset is originally from GEO:GSM605333, whereas H9 dataset is originally from GEO:GSM602292.

#### Comparison with ENCODE ChIP-Seq TF datasets

ENCODE datasets were downloaded through the ENCODE webserver, we compared several cell types, but narrow down to H1 cell line if available (e.g. YY1). For analysis of proximal binding sites with LEUTX we used ‘conservative IDR thresholded’ narrowpeaks bed files and the deepTools Python package as shown below.^85^ The used datasets shown in Figure S4 are: ENCFF563SWF (ARID3A_K562), ENCFF879ZMI (ARID2_K562), ENCFF113BTA (YY1_H1), ENCFF792HJJ (NFRKB_HEK293T), ENCFF786IZD (ZNF462_GM23338), ENCFF970MYF (KLF5_GM12878), ENCFF914NEO (SP2_H1), ENCFF305PPC (SP1_H1), ENCFF532VPN (CREBBP_K562), ENCFF539ZQW (EP300_K562), ENCFF726NGV (EP300_HepG2), ENCFF307PSW (EP300_HepG2), ENCFF899RKF (EP300_K562), ENCFF840MWN (EP300_H1), ENCFF492IMA (SMC3_HepG2), ENCFF289LLT (SMC3_K562), ENCFF532ZYE (RAD21_H1), ENCFF960TEU (RAD21_K562).

First a Dox+ and Dox- subtract was created using bigwigCompare with default settings producing log2ratios for the Dox+ and Dox- subtract files (that are then shown as the y-axis intesity (log2 ratio) in the relevant plots).Then, we used computeMatrix and plotProfile to plot the Dox+ and Dox- subtracts against ENCODE experiment conservative IDR thresholded peak narrowpeaks bed files with settings: referencepoint center, beforeRegionStartLength/ afterRegionStartLength 2500, binsize 50, sortRegions keep, missingDataAsZero, skipZeros.

#### Comparison with embryonic ATAC-seq study

Data from GSE101571 ^44^ was downloaded through NCBI data repository (accessed 21.3.2021), we used bigwig and bed files from the study. We compared them to our ChIP-Seq data using deepTools as shown above. Further, wig files for 4 cell, 8 cell and icm stages and primed hESCs from the same study were converted to tdf and into vector graphics using Integrative Genomics Viewer.^66^ To construct Figure S8E, we further downloaded default datasets Gm06990 CTCF, HUVEC CTCF, and K562 CTCF that are available on the Integrative Genomics Viewer server (https://software.broadinstitute.org/software/igv/).

#### Comparison with 8CLC datasets

Data from recent 8CLC papers^36^^,^^37^^,^^38^ was used in the following way: when discussing ‘hub genes’ we refer to Mazid et al. 2022^36^ Table S5: Full list of 2,162 hub genes and their relative expression level in three cell states. This data was used to construct Figure 4C. When referring to ‘DEG genes between 8CLC and non-8CLC” we refer to Mazid et al. 2022^36^ Table S6. Full list of DEGs between 8CLCs and non-8CLCs in scRNA-seq (droplet-based) of stepwise e4CL-D5 cells filtered for FDR < 0.05. When referring to 8CLC marker genes we are referring to Taubenschmid-Stowers et al. 2022^37^ Table S2. 8CLC signature. 8C-like cell gene expression signature based on single cell RNA-seq of 8CLCs compared to naive hESCs. And finally, when referring to iBM genes we refer to Yoshihara et al. 2022,^38^ Table S3. List of marker genes in each cluster filtered for iBM cluster only. Figure 4A was constructed by interrogating each dataset and displaying all genes upregulated by LEUTX in our STRT-Seq and appearing in at least two of these datasets.

## Data Availability

The datasets generated during this study are available at: STRT-Seq fastq and BAM files have been deposited to EMBL/EBI ArrayExpress E-MTAB-10539, NET-CAGE fastq and BAM files have been deposited to ENA PRJEB45266, ChIP-Seq fastq and BAM files have been deposited to ENA PRJEB45266, Proteomics Raw Spectral Files and Search Files deposited to MassIVE MSV000087381. This paper does not report original code. Additional information required to reanalyze the data reported in this paper is available from the lead contact upon request.

## Supplemental information

Document S1. Figures S1–S11

Table S1. Proteomics results related to Figure 1 Statistically and contaminant filtered SAINTexpress (BFDR < 0.05) output list.txt files, combining both AP-MS and BioID data (labeled: Detection) for LEUTX, LEUTX 9aaTAD mutant, and K57A mutant. These files were used to perform interactome and enrichment analysis.

Table S2. CORUM Enrichment related to Figure 1 Enrichment of Prey proteins in CORUM protein complex database compared to total lysate control in all proteomics datasets.

Table S3. Expression of Protein-protein interactions in embryonic data related to Figure 1 Expression values of identified protein-protein interactions in cleavage stage embryo transcriptomics dataset ^[3]^. 129 identified preys were found expressed in at least one timepoint between 2-cell and morulae stages ^[3]^.

Table S4. Identified upregulated NET-CAGE Enhancers related to Figure 2 1990 upregulated (FDR < 0.05, logFC>0) enhancer locations (hg19).

Table S5. Identified upregulated NET-CAGE Promoters related to Figure 2 2664 upregulated (FDR < 0.05, logFC>0) promoter locations (hg19).

Table S6. Identified differential ChIP-Seq HA peaks (MACS2 output file) related to Figure 2 4962 differential (FDR < 0.05) ChIP-Seq peaks (GRCh38). Hg19 liftover in second tab.

Table S7. SpaMo analysis related to Figure 2 Spaced Motif analysis tool SpaMo (MEME Suite) results tabled per genomic dataset. SpaMo identifies enriched motifs around a searched motif. We searched for the EEA-motif in our four genomic datasets (Fasta of genomic locations of upregulated NET-CAGE enhancers and promoters, ChIP-Seq peaks, and STRT TFEs). In this table, significantly (SpaMo E-value) enriched motifs are tabled per dataset.

Table S8. Comparison of repetitive element overlap of LEUTX NET-CAGE results to FANTOM5 results related to Figure 3 Chi-squared test comparing NET-CAGE enhancers to FANTOM5 enhancers and NET-CAGE promoters to FANTOM5 CAGE promoters. Comparison of repetitive element category overlap (includes all overlaps, some enhancers overlap more than one repetitive element) between LEUTX NET-CAGE results and FANTOM5 Enhancers and Promoters.

Table S9. HOMER analysis related to Figure 3 HOMER annotate peaks genome feature association analysis output for ChIP-Seq peaks. These results reflect possible genomic annotations genome wide, including repetitive elements.

Table S10. STRT DE Gene FoldChanges tabled per timepoint related to Figure 3 Log2FoldChanges for each detected differentially expressed gene listed in each timepoint.

Table S11. Gawriyski et al. - Jouhilahti et al. (2016) overlap related to Figure 3 Overlap between sequencing results for LEUTX overexpression in Jouhilahti et al. (2016) ^[8]^ and STRT results produced in this paper.

Table S12. LEUTX cloning oligos, promoter and enhancer guide oligos and qPCR oligos related to Figure 4 and STAR Methods

Table S13. MODEL OF KIX domain in complex of 9aaTAD related to Figure 1 and STAR Methods
