# Supplementary material for: Comprehensive characterization of the embryonic factor LEUTX
Source: iScience. 2023 Feb 9;26(3):106172. doi: 10.1016/j.isci.2023.106172 (PMC9978639; doi:10.1016/j.isci.2023.106172)
Supplement: Document S1. Figures S1–S11 [file mmc1.pdf]

iScience, Volume *26*

Supplemental information

Comprehensive characterization of the embryonic factor LEUTX

Lisa Gawriyski, Eeva-Mari Jouhilahti, Masahito Yoshihara, Liangru Fei, Jere Weltner, Tomi T. Airenne, Ras Trokovic, Shruti Bhagat, Mari H. Tervaniemi, Yasuhiro Murakawa, Kari Salokas, Xiaonan Liu, Sini Miettinen, Thomas R. Bürglin, Biswajyoti Sahu, Timo Otonkoski, Mark S. Johnson, Shintaro Katayama, Markku Varjosalo, and Juha Kere

Supplemental information titles and legends

Supplemental Figures

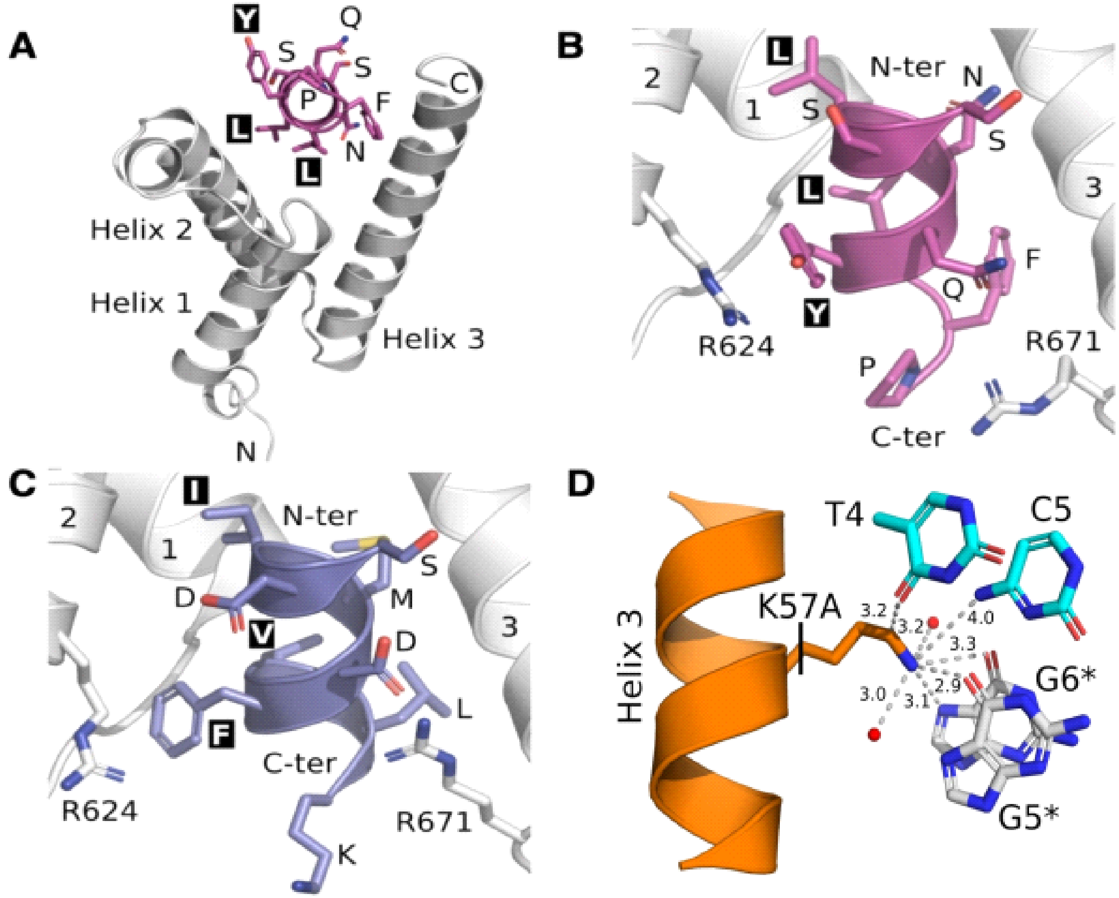

Figure S1. Structural modeling of LEUTX mutants related to Figure 1A.

A) LEUTX ^{178}SSLNQYLFP^{186} 9aaTAD (magenta) modelled onto the KIX domain structure.

B) Close-up of (a)

C) Close-up of the mixed-lineage leukemia (MLL) 9aaTAD (blue) bound to the KIX domain; from the NMR structure of the complex (PDB code 2LXT).

D) K57A homeodomain mutation is predicted to lose binding affinity through the loss of multiple predicted interactions (dotted lines, distances in Å) with the DNA motif and water molecules (red spheres).

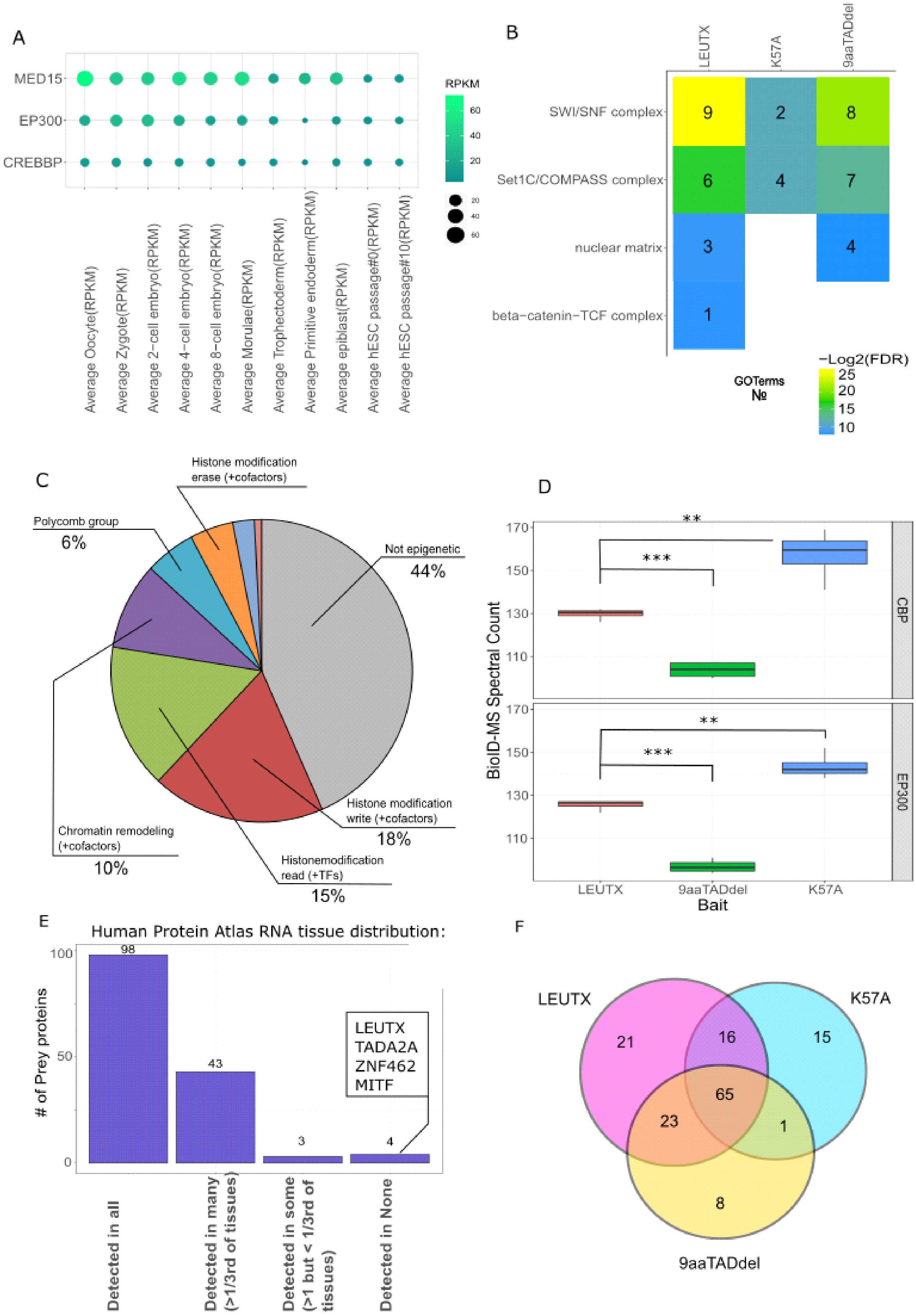
A
MED15
EP300
CREBBP
RPKM
60
40
20
Average Oocyte(RPKM)
Average Zygote(RPKM)
Average 2-cell embryo(RPKM)
Average 4-cell embryo(RPKM)
Average 8-cell embryo(RPKM)
Average Morulae(RPKM)
Average Trophectoderm(RPKM)
Average Primitive endoderm(RPKM)
Average epiblast(RPKM)
Average hESC passage#0(RPKM)
Average hESC passage#10(RPKM)
B
LEUTX
K57A
9aaTADdel
SWI/SNF complex
9
2
8
Set1C/COMPASS complex
6
4
7
nuclear matrix
3
4
beta-catenin-TCF complex
1
GOTerms №
-Log2(FDR)
25
20
15
10
C
Histone modification erase (+cofactors)
Polycomb group 6%
Not epigenetic 44%
Histone modification write (+cofactors) 18%
Chromatin remodeling (+cofactors) 10%
Histonemodification read (+TFs) 15%
D
BioID-MS Spectral Count
CBP
EP300
LEUTX
9aaTADdel
K57A
Bait
E
Human Protein Atlas RNA tissue distribution:
# of Prey proteins
98
43
3
4
LEUTX
TADA2A
ZNF462
MITF
Detected in all
Detected in many (>1/3rd of tissues)
Detected in some (>1 but < 1/3rd of tissues)
Detected in None
F
LEUTX
K57A
9aaTADdel
21
16
15
65
23
1
8

Figure S2. Proteomics supplements related to Figure 1

A) Expression of KIX-domain containing co-activator proteins MED15, EP300 and CBP in human embryonic and hESC data from Yan et al. (2013) [1].

B) Gene Ontology: Cellular Compartments heatmap comparison for LEUTX and the two mutants. LEUTX is strongly localized to a few chromatin modifying complexes, such as the SWI/SNF complex (BAF complex). The K57A mutant seems to be displaced from the nuclear matrix. Colors depict Log2 FDR of the lowest FDR GO term (FDR < 0.05), and numbers depict the number of collapsed GO terms under the higher order term.

C) Annotation of LEUTX preys to EpiFactors database. In total 70 proteins (54%) in the LEUTX interactome were listed as having epigenetic or chromatin modification function in the EpiFactors database [2]. Most enriched functions were related to histone modification, the most common being writing histone modifications (22 proteins, 18% of interactome).

D) The spectral counts of all replicates in BioID-MS data of each bait plotted for CBP and EP300. In both CBP and EP300, the spectral counts are statistically significantly lower in 9aaTADdel samples than in the LEUTX samples (Student's t-test, $p < 0.001$). Conversely, in K57A samples EP300 and CBP values are significantly higher than in LEUTX samples (Student's T-test, $p < 0.01$). P-values labeled ($p < 0.5$ = *, $p < 0.01$ = **, $p < 0.001$ < ***).

E) RNA level tissue expression of all (149) detected preys in the Human Protein Atlas database [3]. Majority of detected preys are general factors detected in all tissues (98, 66%). Only four factors were not detected expressed in any tissues, LEUTX itself, TADA2A, ZNF462 and MITF.

F) Venn diagram of all (149) of the detected protein-protein interactions, depicting how many of the interactions are shared. Majority of interactions are shared by all (65, 44%) or shared between LEUTX and either one of the mutants. Fairly few interactors are only detected in the mutants.

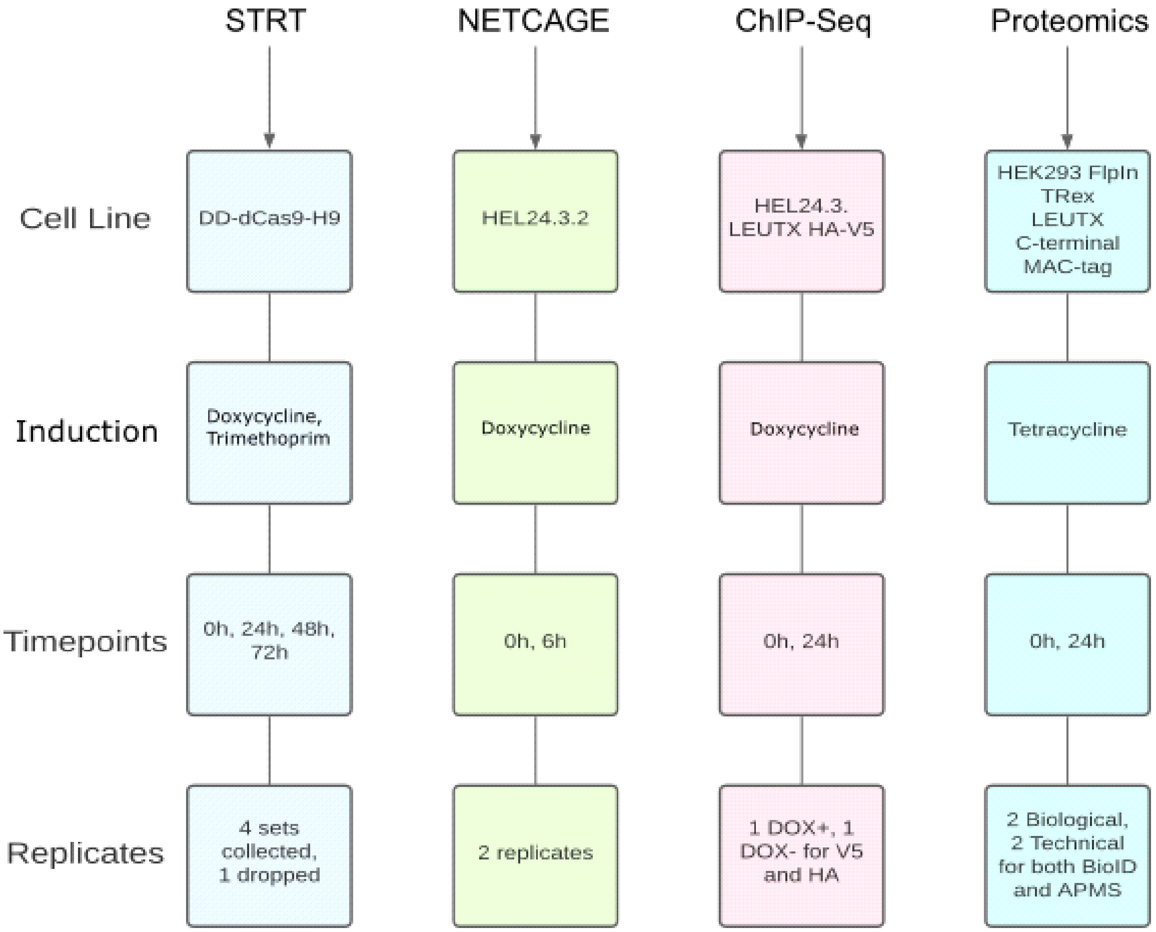

Figure S3. Overview of the methods and experimental details related to Figure 2

The cell lines, induction, timepoints and the number of replicates for every experiment. For STRT, NET-CAGE, and ChIP-Seq we used pluripotent stem cell models. For affinity purification. which requires a massive number of cells to be grown, we used the HEK293 cell line.

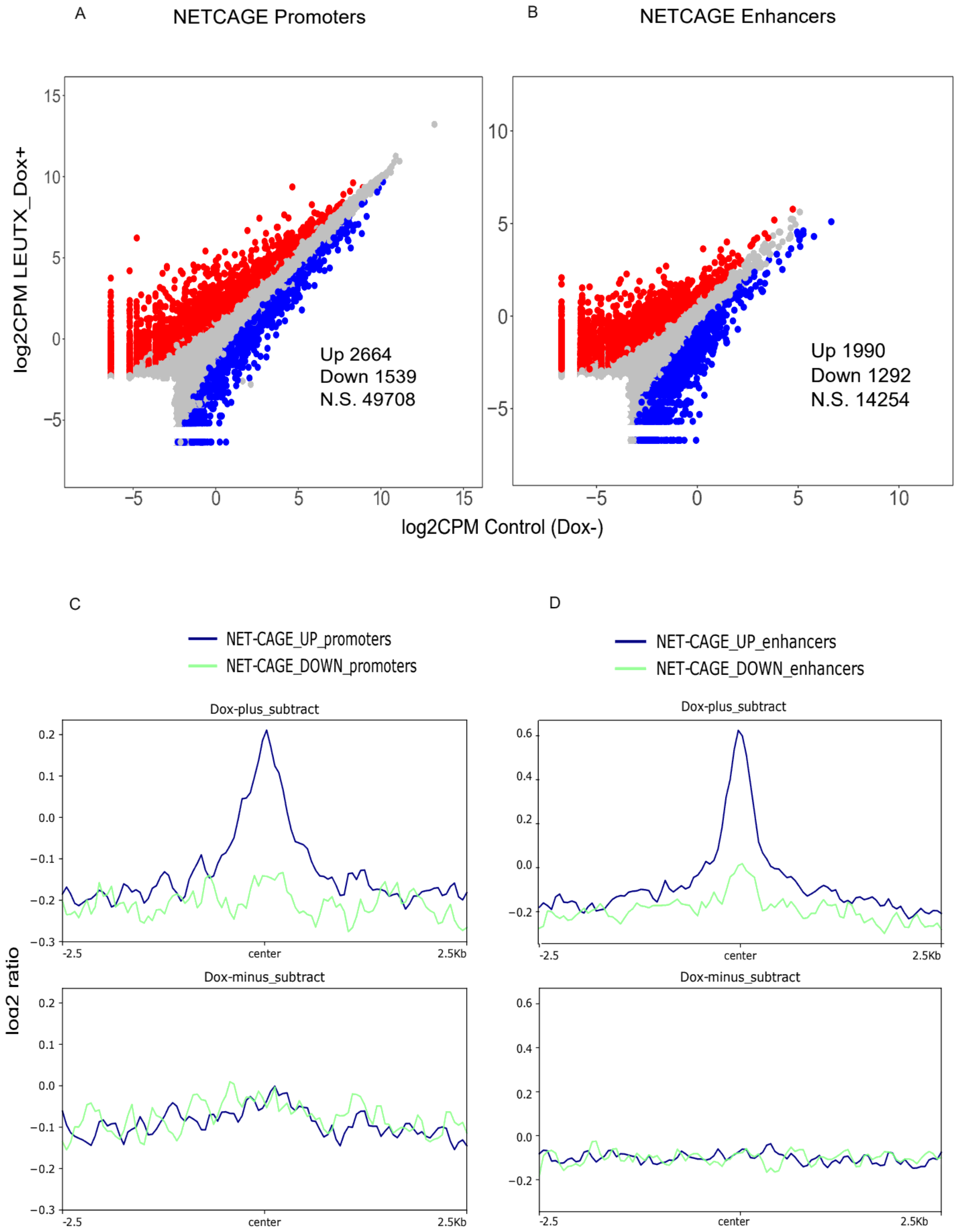
A
NETCAGE Promoters
B
NETCAGE Enhancers
log2CPM LEUTX_Dox+
Up 2664
Down 1539
N.S. 49708
Up 1990
Down 1292
N.S. 14254
log2CPM Control (Dox-)
C
NET-CAGE_UP_promoters
NET-CAGE_DOWN_promoters
D
NET-CAGE_UP_enhancers
NET-CAGE_DOWN_enhancers
Dox-plus_subtract
Dox-minus_subtract
log2 ratio
-2.5
center
2.5Kb

Figure S4. NET-CAGE supplements related to Figure 2

Comparison of LEUTX expressing (Dox+, 2 replicates) and Control (Dox-, 2 replicates) NET CAGE data. Promoters and Enhancers with average expression < -2.5 log2CPM were filtered out. Differential expression was calculated through EdgeR Generalized Linear Model Likelihood Ratio Test and upregulation was defined as log2FC > 0, whereas downregulation as log2FC < 0.

A) NET-CAGE Promoter scatter plot. By NET-CAGE we identified 2664 upregulated and 1539 downregulated promoters.

B) NET-CAGE Enhancer scatter plot. By NET-CAGE we identified 1990 upregulated and 1292 downregulated enhancers.

C) ChIP-Seq Dox plus subtract overlayed with promoters. LEUTX ChIP-Seq peaks preferably overlap upregulated promoters.

D) ChIP-Seq Dox plus subtract overlayed with enhancers. LEUTX ChIP-Seq peaks preferably overlap upregulated enhancers.

For b and c genome is partitioned into bins of equal size, and then reads are counted per bin. Y-axis is the log2 ratio of number of NET-CAGE reads per bin between the Dox+ and Dox-subtracts of ChIP-Seq samples, whereas the x-axis is distance from center of ChIP-Seq peaks.

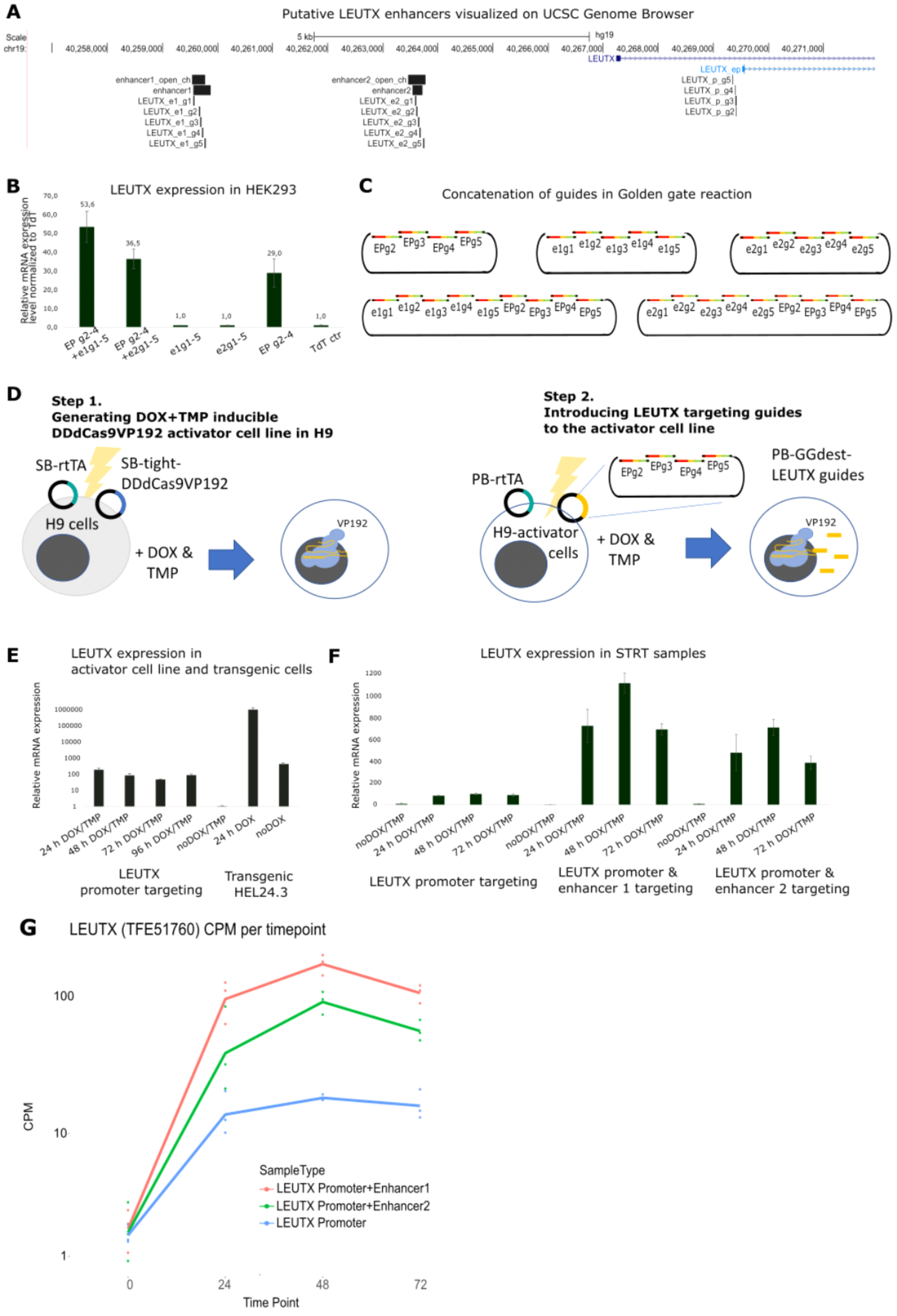
A
Putative LEUTX enhancers visualized on UCSC Genome Browser
Scale
chr19:
5 kb
hg19
40,258,000
40,259,000
40,260,000
40,261,000
40,262,000
40,263,000
40,264,000
40,265,000
40,266,000
40,267,000
40,268,000
40,269,000
40,270,000
40,271,000
LEUTX
LEUTX_ep
enhancer1_open_ch
enhancer1
LEUTX_e1_g1
LEUTX_e1_g2
LEUTX_e1_g3
LEUTX_e1_g4
LEUTX_e1_g5
enhancer2_open_ch
enhancer2
LEUTX_e2_g1
LEUTX_e2_g2
LEUTX_e2_g3
LEUTX_e2_g4
LEUTX_e2_g5
LEUTX_p_g5
LEUTX_p_g4
LEUTX_p_g3
LEUTX_p_g2
B
LEUTX expression in HEK293
Relative mRNA expression level normalized to TdT
53,6
36,5
1,0
1,0
29,0
1,0
EP g2-4 +e1g1-5
EP g2-4 +e2g1-5
e1g1-5
e2g1-5
EP g2-4
TdT ctr
C
Concatenation of guides in Golden gate reaction
EPg2 EPg3 EPg4 EPg5
e1g1 e1g2 e1g3 e1g4 e1g5
e2g1 e2g2 e2g3 e2g4 e2g5
e1g1 e1g2 e1g3 e1g4 e1g5 EPg2 EPg3 EPg4 EPg5
e2g1 e2g2 e2g3 e2g4 e2g5 EPg2 EPg3 EPg4 EPg5
D
Step 1.
Generating DOX+TMP inducible DDdCas9VP192 activator cell line in H9
SB-rtTA
SB-tight-DDdCas9VP192
H9 cells
+ DOX & TMP
VP192
Step 2.
Introducing LEUTX targeting guides to the activator cell line
PB-rtTA
EPg2 EPg3 EPg4 EPg5
PB-GGdest-LEUTX guides
H9-activator cells
+ DOX & TMP
VP192
E
LEUTX expression in activator cell line and transgenic cells
Relative mRNA expression
24 h DOX/TMP
48 h DOX/TMP
72 h DOX/TMP
96 h DOX/TMP
noDOX/TMP
24 h DOX
noDOX
LEUTX promoter targeting
Transgenic HEL24.3
F
LEUTX expression in STRT samples
Relative mRNA expression
noDOX/TMP
24 h DOX/TMP
48 h DOX/TMP
72 h DOX/TMP
LEUTX promoter targeting
LEUTX promoter & enhancer 1 targeting
LEUTX promoter & enhancer 2 targeting
G
LEUTX (TFE51760) CPM per timepoint
CPM
100
10
1
0
24
48
72
Time Point
SampleType
LEUTX Promoter+Enhancer1
LEUTX Promoter+Enhancer2
LEUTX Promoter

Figure S5. Validation of putative LEUTX enhancers and generation of DD-dCas9VP192 inducible cell line related to STAR methods

A) LEUTX promoter and putative enhancer regions predicted from TetOn DUX4 hESC NET-CAGE dataset[4] together with designed CRISPR guide-RNAs are visualized on UCSC genome browser.

B) Expression level of LEUTX in HEK293 cells after co-transfection of LEUTX promoter and enhancer targeting guides and dCas9 activator with VP192 domain as measured by qPCR. Figure shows the effect of putative enhancer targeting guide RNAs in comparison to the promoter targeting guide RNAs only. EP g2-4, LEUTX embryonic promoter guides 2-4; e1g1-5, enhancer1 guides 1-5; e2g1-5, enhancer2 guides 1-5; TDT, tdTomoto control.

C) Concatenation of validated enhancer and promoter targeting guides in a GoldenGate reaction as described in (Balboa et al. 2015) [5] Guide cassettes containing both promoter and enhancer guides was further cloned together. Finally, the guide cassettes were cloned to piggyBac vector.

D) Generation of an inducible DDdCas9VP192 activator cell line using Sleeping beauty transposition system and further integration of validated LEUTX enhancer and promoter targeting guides by PiggyBac transposition as exemplified by integration of 4 LEUTX promoter targeting guides.

E) Confirmation of LEUTX activation upon Doxycycline and trimethoprim treatment in endogenously activated and transgenic cells by qPCR.

F) Confirmation of the Cas9 and LEUTX expression in all samples collected for the STRT-Seq experiment.

G) Comparison of the LEUTX STRT TFE counts per million (CPM) in different sample types (LEUTX promoter only, LEUTX promoter and enhancer 1, LEUTX promoter and enhancer 2) in STRT-Seq samples.

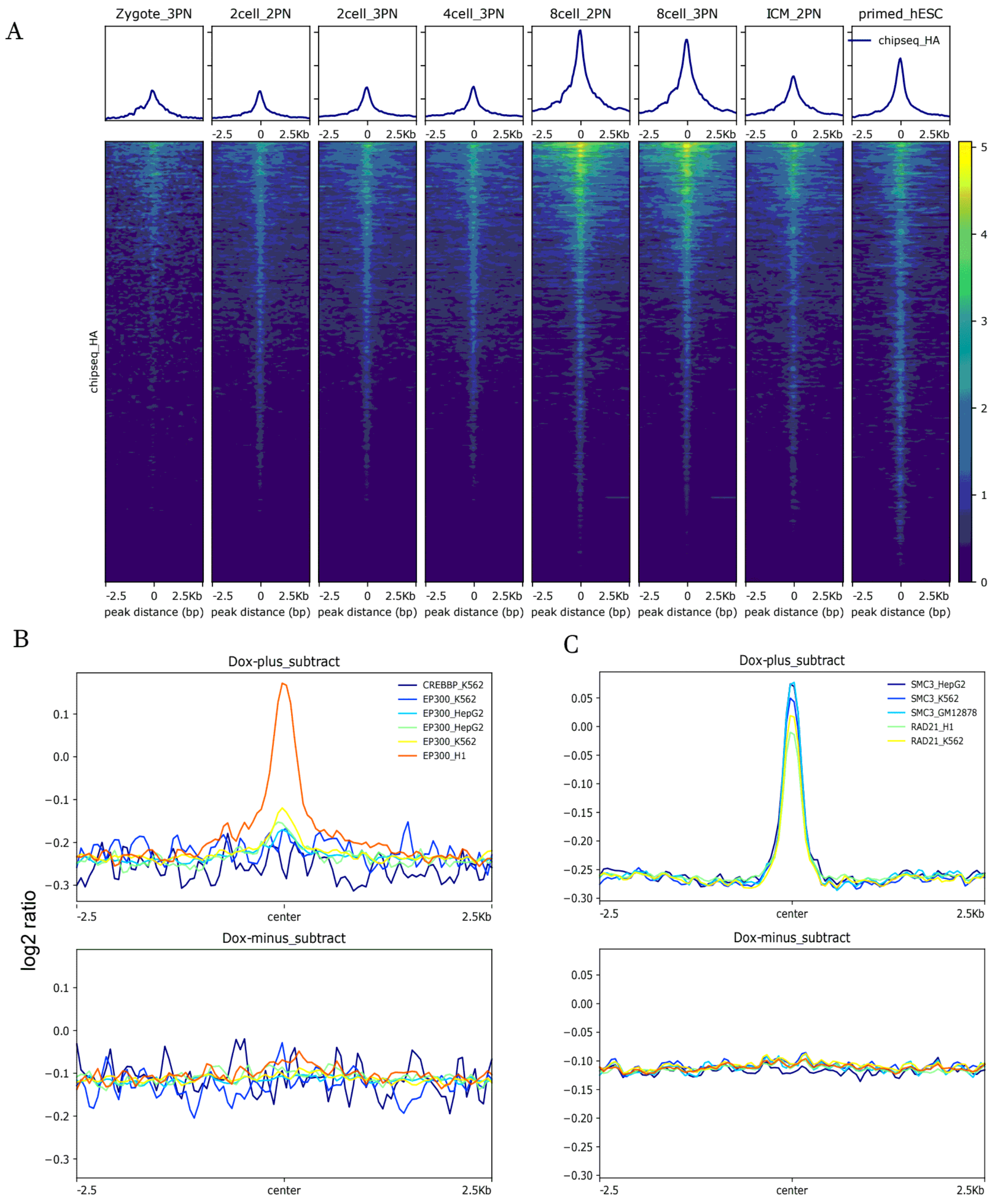

Figure S6. Comparative analysis of LEUTX ChIP-Seq data to Wu et al. (2018) embryonic ATAC-seq data related to Figure 2

A) The heatmap visualizes the enrichment of LEUTX binding sites around accessible open chromatin regions. Comparing LEUTX associated differential ChIP-Seq peaks to the accessible open regions in different embryonic cell types[6] we find highest intensity in the 8-cell samples, which corresponds with LEUTX expression window in the preimplantation embryo. Y-axis of the heatmap represents the LEUTX binding sites per genomic locations ordered by the enrichment score and x-axis shows the distance from the center of ATAC-seq peaks. Color indicates the enrichment score. The profile plot on top of each heatmap summarizes the LEUTX ChIP-Seq read density across all ATAC-seq peaks.

B) LEUTX ChIP-Seq subtract overlayed with EP300 TF ChIP-Seq peaks. EP300 binding sites notably overlap with Dox+ ChIP-Seq sites in the H1 cell line, but not as strongly in the cancer cell lines. Y-axis is the log2 ratio of the Dox+ subtract, whereas the x-axis is distance from center of peaks.

C) Cohesin complex members SMC3 and RAD21 identified as dynamic interactors of LEUTX through BioID-MS are also often proximal in LEUTX binding sites. SMC3 shows little variation between cell lines. Y-axis is the log2 ratio of the Dox+ subtract, whereas the x-axis is distance from center of peaks.

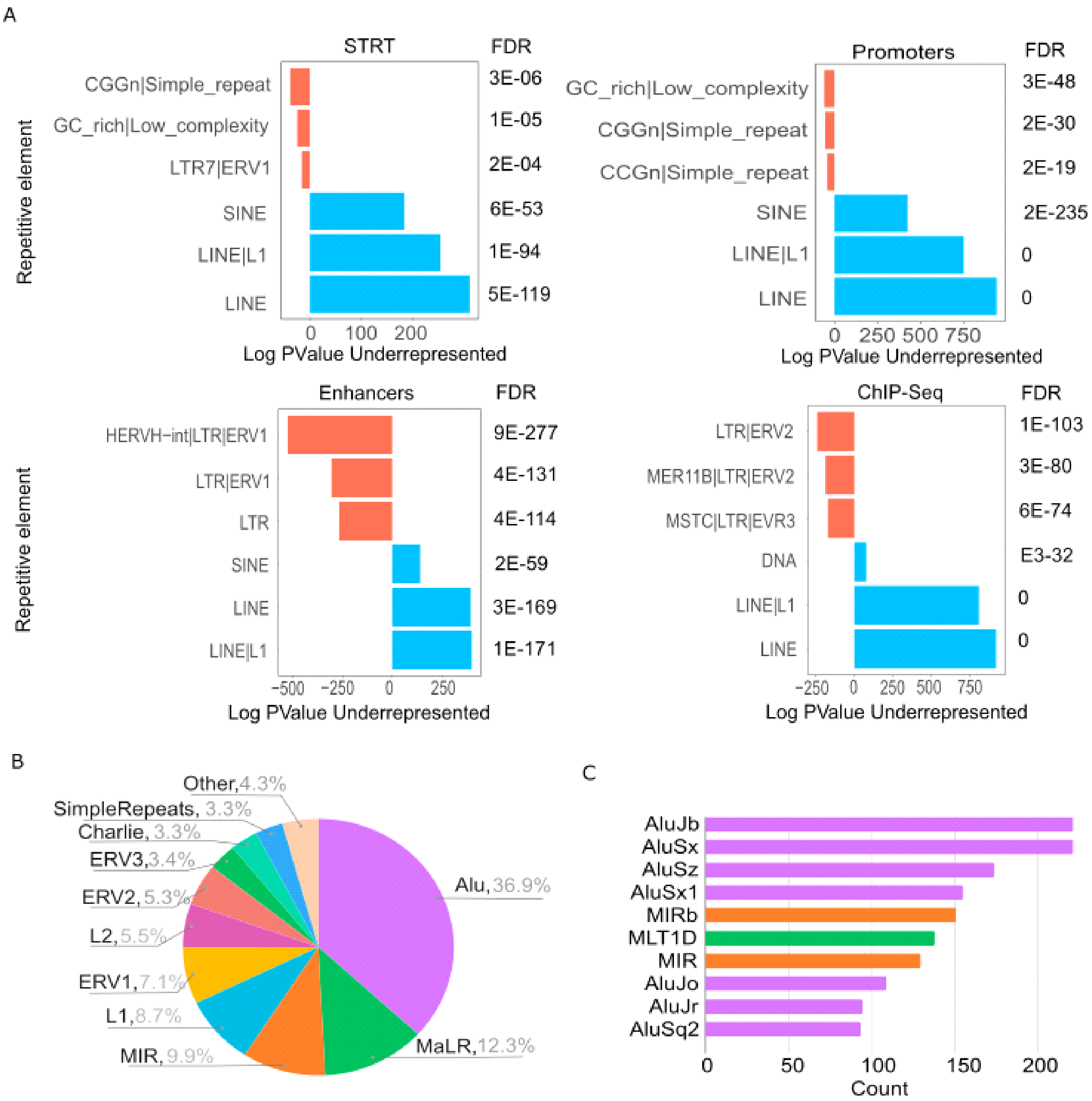

Figure S7. Repetitive elements supplements related to Figure 3

A) HOMER repetitive element enrichment analysis for the key data types in our study. Upregulated STRT-Seq TFEs, upregulated enhancers, upregulated promoters, and ChIP-Seq peaks are compared to genomic frequency to produce estimates of under- or over enrichment. Overrepresentation is shown as red bars growing in the negative direction (Log PValue Underrepresented), whereas underrepresentation is shown as blue bars growing in positive direction.

B) The pie chart shows the percentages of the repetitive element subgroups in LEUTX ChIP-Seq binding sites overlapping with all repetitive elements.

C) The counts of most common repetitive element subtypes in LEUTX binding sites, identified through ChIP-Seq.

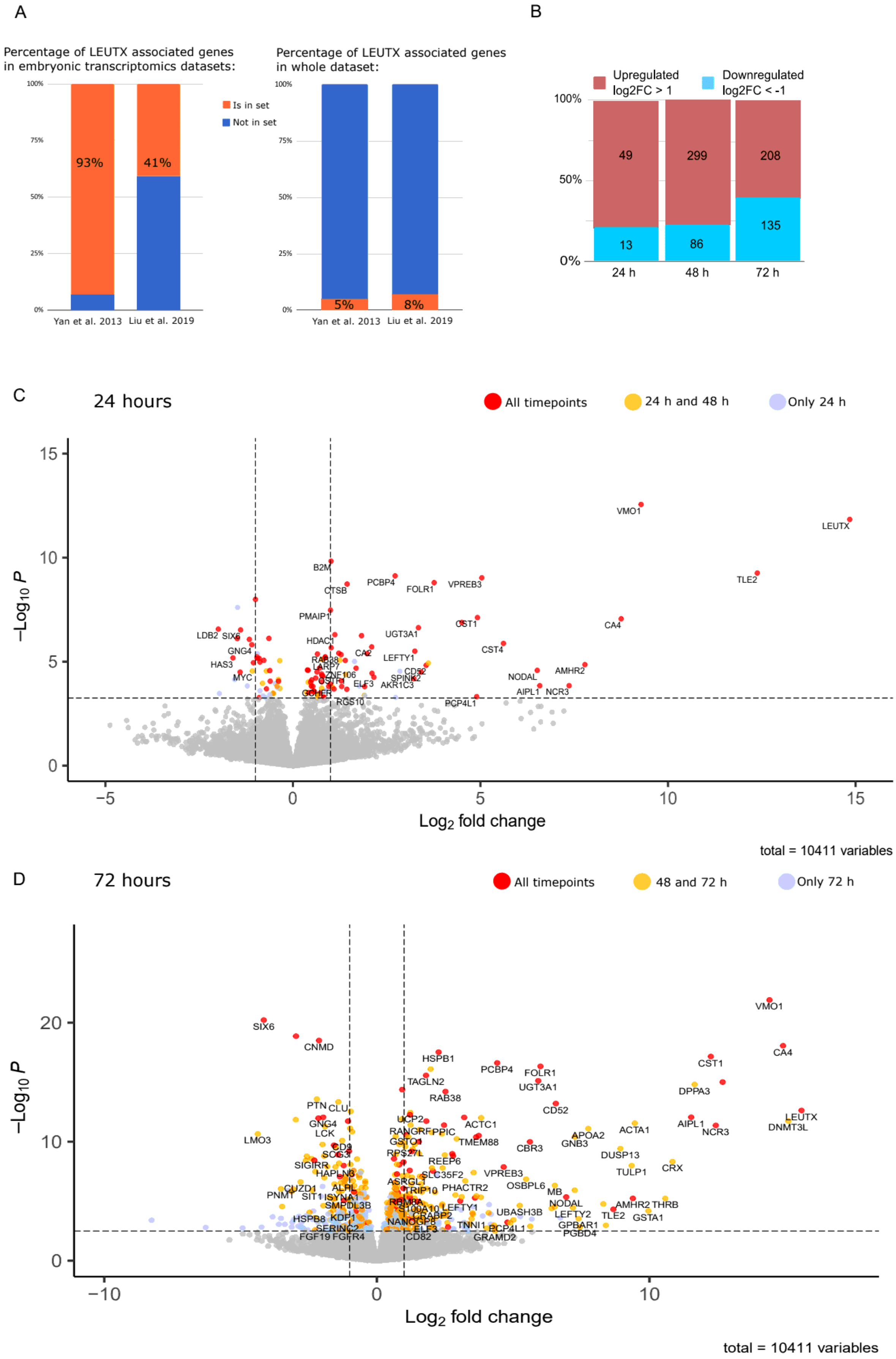
A
Percentage of LEUTX associated genes in embryonic transcriptomics datasets:
100%
75%
50%
25%
0%
93%
41%
Yan et al. 2013
Liu et al. 2019
Is in set
Not in set
Percentage of LEUTX associated genes in whole dataset:
5%
8%
B
Upregulated log2FC > 1
Downregulated log2FC < -1
49
299
208
13
86
135
24 h
48 h
72 h
C
24 hours
All timepoints
24 h and 48 h
Only 24 h
−Log10 P
Log2 fold change
VMO1
LEUTX
TLE2
B2M
PCBP4
FOLR1
VPREB3
CTSB
PMAIP1
CA4
CST1
UGT3A1
CST4
LDB2
SIX6
HDAC1
GNG4
CA2
LEFTY1
HAS3
RAB38
LARP7
CD52
AMHR2
NODAL
ZNF106
SPINK2
MYC
GSTP1
ELF3
AKR1C3
AIPL1
NCR3
COHER
RGS10
PCP4L1
total = 10411 variables
D
72 hours
All timepoints
48 and 72 h
Only 72 h
VMO1
SIX6
CA4
CNMD
HSPB1
CST1
PCBP4
FOLR1
TAGLN2
UGT3A1
DPPA3
RAB38
PTN
CLU
CD52
LEUTX
AIPL1
DNMT3L
UCP2
GNG4
ACTC1
NCR3
ACTA1
LCK
RANGRF
PPIC
APOA2
LMO3
GSTO1
TMEM88
GNB3
CBR3
CD9
SOCS3
RPS27L
DUSP13
REEP6
SIGIRR
CRX
HAPLN3
TULP1
SLC35F2
VPREB3
ASRGL1
OSBPL6
CUZD1
ALPL
TRIP10
PHACTR2
MB
PNMT
SIT1
ISYNA1
NODAL
AMHR2
THRB
SMPDL3B
LEFTY1
UBASH3B
LEFTY2
TLE2
GSTA1
HSPB8
KDF1
CRABP2
NANOGP8
GPBAR1
SERINC2
ELF3
TNNI1
PCP4L1
PGBD4
FGF19
FGFR4
CD82
GRAMD2
total = 10411 variables

Figure S8. LEUTX STRT time point volcano plots related to Figure 3

A) Percentage of genes expressed in two embryonic transcriptomics studies Yan et al. (2013) [1] and Liu et al. (2019) [7]. First shown as percentage of total number of differentially expressed genes identified through LEUTX STRT that are also expressed in said transcriptomics studies. 93% of LEUTX associated genes identified through STRT are found expressed in Yan et al. (2013) [1] and 41% of LEUTX associated genes identified through STRT and found expressed in Liu et al. (2019) [7]. Then, shown as the percentage of LEUTX associated genes in the whole datasets, 5% of Yan et al. (2013) [1] are LEUTX associated genes and 8% from Liu et al. (2019) [7] respectively.

B) Number of significantly upregulated and downregulated genes in each timepoint. Significant upregulation defined as log2FC > 1 (shown in red), and downregulation as log2FC <-1 (shown in blue). Number of genes matching the criteria are shown per timepoint.

C) Volcano plot of the genes up- and downregulated at 24 hours shows strong immediate upregulation of several terms that remain upregulated throughout all the timepoints (red color). Low downregulation of a few targets, such as MYC and SIX6 can also be seen. Genes only up- or downregulated in the 24 h time point are shown in light blue, those only in the 24 and 48 h timepoints in orange.

D) Volcano plot of the genes up- and downregulated at 72 hours. Like in the 48h timepoint, upregulation remains strong but downregulation weak. Many terms upregulated in 48h remain so in 72 h (orange color). Genes up- or downregulated in all timepoints shown in red, those only up or downregulated in the 72 h time point shown in light blue.

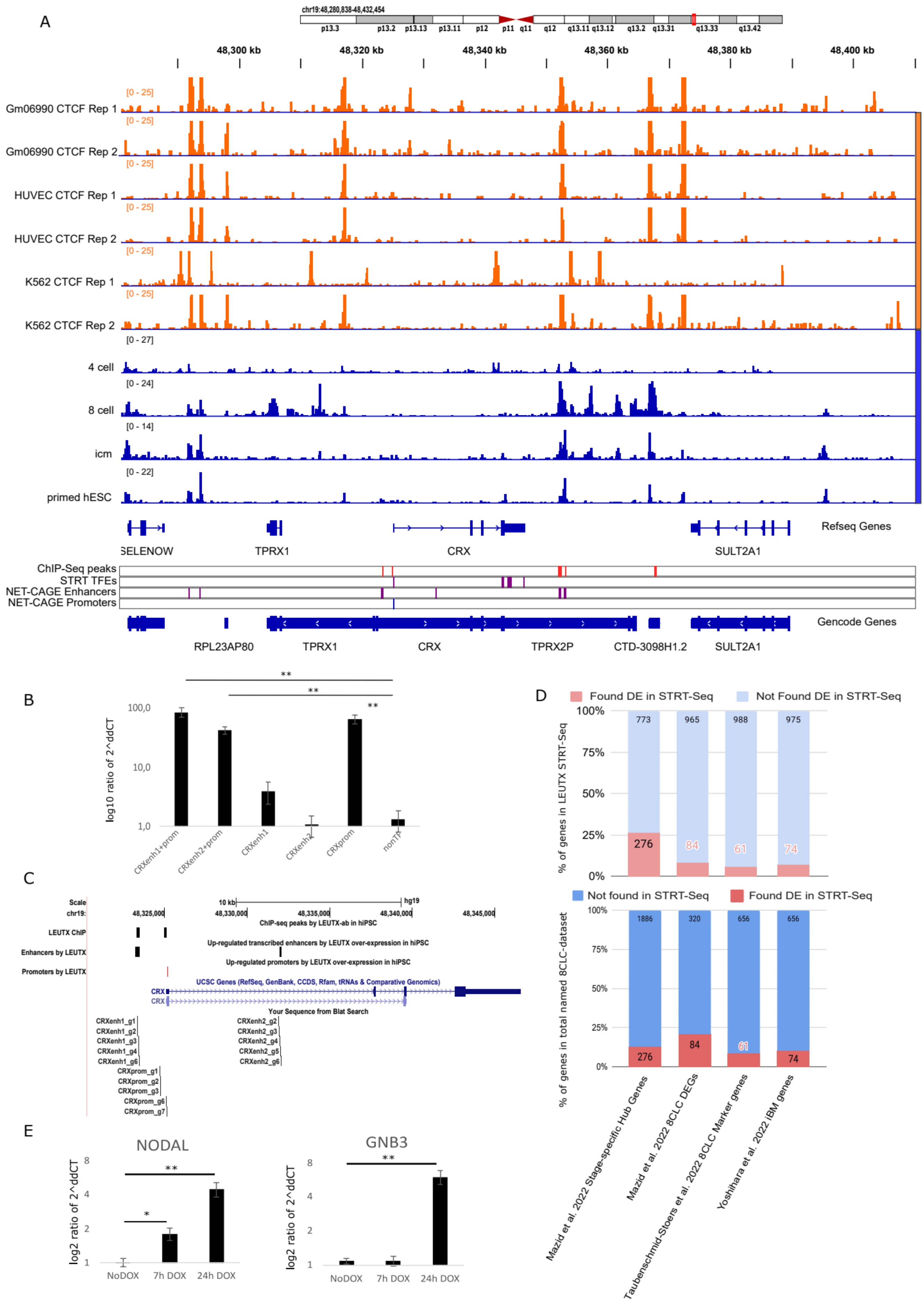
A
chr19:48,280,838-48,432,454
p13.3
p13.2
p13.13
p13.11
p12
p11
q11
q12
q13.11
q13.12
q13.2
q13.31
q13.33
q13.42
48,300 kb
48,320 kb
48,340 kb
48,360 kb
48,380 kb
48,400 kb
[0 - 25]
Gm06990 CTCF Rep 1
Gm06990 CTCF Rep 2
HUVEC CTCF Rep 1
HUVEC CTCF Rep 2
K562 CTCF Rep 1
K562 CTCF Rep 2
[0 - 27]
4 cell
[0 - 24]
8 cell
[0 - 14]
icm
[0 - 22]
primed hESC
Refseq Genes
SELENOW
TPRX1
CRX
SULT2A1
ChIP-Seq peaks
STRT TFEs
NET-CAGE Enhancers
NET-CAGE Promoters
Gencode Genes
RPL23AP80
TPRX1
CRX
TPRX2P
CTD-3098H1.2
SULT2A1
B
log10 ratio of 2^ddCT
100,0
10,0
1,0
**
**
**
CRXenh1+prom
CRXenh2+prom
CRXenh1
CRXenh2
CRXprom
nonTP
C
Scale
chr19:
10 kb
hg19
48,325,000
48,330,000
48,335,000
48,340,000
48,345,000
ChIP-seq peaks by LEUTX-ab in hiPSC
LEUTX ChIP
Up-regulated transcribed enhancers by LEUTX over-expression in hiPSC
Enhancers by LEUTX
Up-regulated promoters by LEUTX over-expression in hiPSC
Promoters by LEUTX
UCSC Genes (RefSeq, GenBank, CCDS, Rfam, tRNAs & Comparative Genomics)
CRX
CRX
Your Sequence from Blat Search
CRXenh1_g1
CRXenh1_g2
CRXenh1_g3
CRXenh1_g4
CRXenh1_g6
CRXprom_g1
CRXprom_g2
CRXprom_g3
CRXprom_g6
CRXprom_g7
CRXenh2_g2
CRXenh2_g3
CRXenh2_g4
CRXenh2_g5
CRXenh2_g6
D
Found DE in STRT-Seq
Not Found DE in STRT-Seq
% of genes in LEUTX STRT-Seq
100%
75%
50%
25%
0%
773
965
988
975
276
84
61
74
Not found in STRT-Seq
Found DE in STRT-Seq
% of genes in total named 8CLC-dataset
100%
75%
50%
25%
0%
1886
320
656
656
276
84
61
74
Mazid et al. 2022 Stage-specific Hub Genes
Mazid et al. 2022 8CLC DEGs
Taubenschmid-Stoers et al. 2022 8CLC Marker genes
Yoshihara et al. 2022 iBM genes
E
NODAL
log2 ratio of 2^ddCT
8
4
2
1
**
*
NoDOX
7h DOX
24h DOX
GNB3
log2 ratio of 2^ddCT
8
4
2
1
**
NoDOX
7h DOX
24h DOX

Figure S9. LEUTX STRT-Seq supplements related to Figure 3

A) CRX genomic locus including PRDL gene family members TPRX1 and TPRX2. Upregulated regulatory regions (NET-CAGE enhancer, NET-CAGE promoter, STRT-Seq TFE) and ChIP-Seq LEUTX binding sites are shown as vertical bars at the bottom of the figure. CTCF binding sites are shown in orange) embryonic ATAC-Seq data[6] is shown in blue. Annotation tracks showing both Refseq and Gencode genes included.

B) Validation of LEUTX-activated transcribed putative *CRX* enhancer. Graph shows CRX expression level relative to non-transfected cells (n=3 transfections). Y-axis shows the log2 ratio of -2^ddCT. Data in are shown as mean ± SEM and p-values were calculated using two-tailed Student's t-test, p-values labeled ($p < 0.5$ = *, $p < 0.01$ = **, $p < 0.001$ < ***).

C) UCSC genome browser view of *CRX* locus. *LEUTX* driven enhancers and promoters by NET-CAGE and LEUTX binding sites are marked as vertical bars above the *CRX* transcript and the guide positions of *CRX* enhancer and promoter targeting guides are marked below the *CRX* transcript.

D) Overlap between our STRT-Seq data and 8CLC paper datasets. Number of differentially expressed (DE) genes that are also detected in the 8CLC papers as portion of the genes identified through STRT-Seq and as a portion of the total studied 8CLC datasets.

E) Further RT-qPCR validation of upregulated genes identified through STRT-seq. Data in are shown as mean ± SEM and p-values were calculated using two-tailed Student's t-test, p-values labeled ($p < 0.5$ = *, $p < 0.01$ = **, $p < 0.001$ < ***).

A

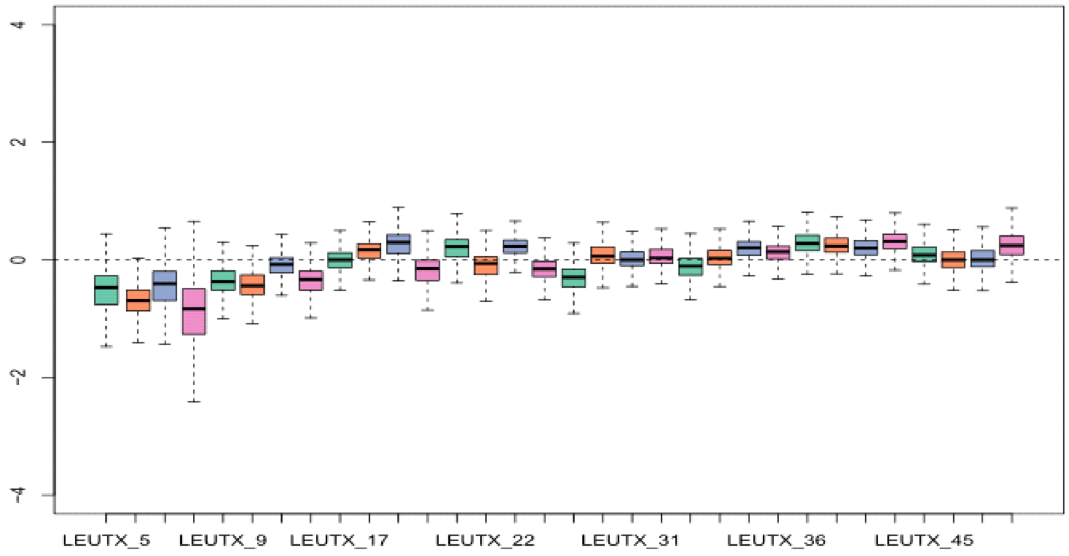

B

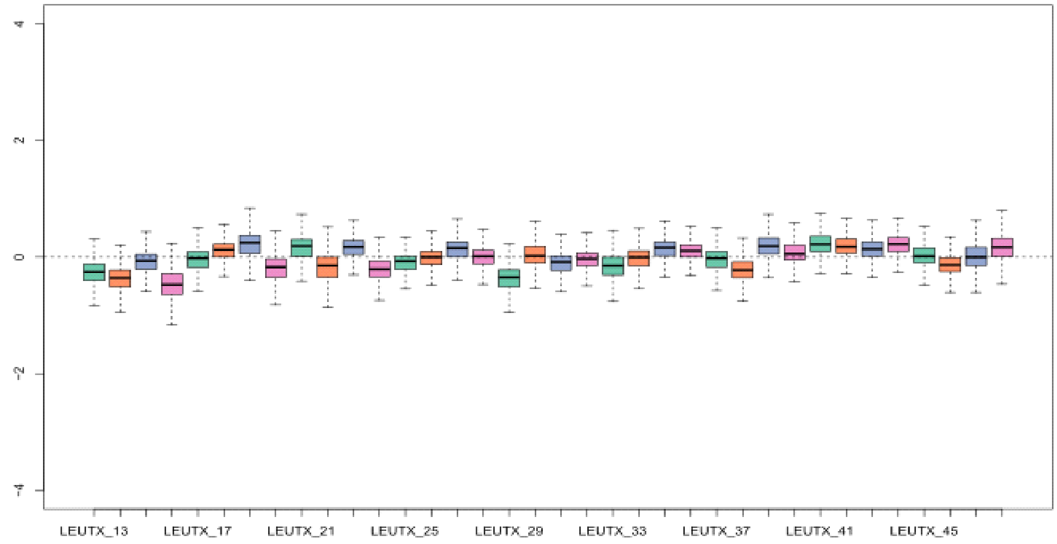

Figure S10. STRT data normalization related to STAR methods

A) Relative Log Expression (RLE) plot of all samples. We observed that the first row on the PCR plate (first 8 samples), were separating from the other samples in QC plots.

B) RLE plot of samples kept in the experiment (13-48). To keep the sample types in the same amounts (e.g. promoter only, promoter + enhancer 1, promoter + enhancer 2) we removed the first twelve samples from the experiment.

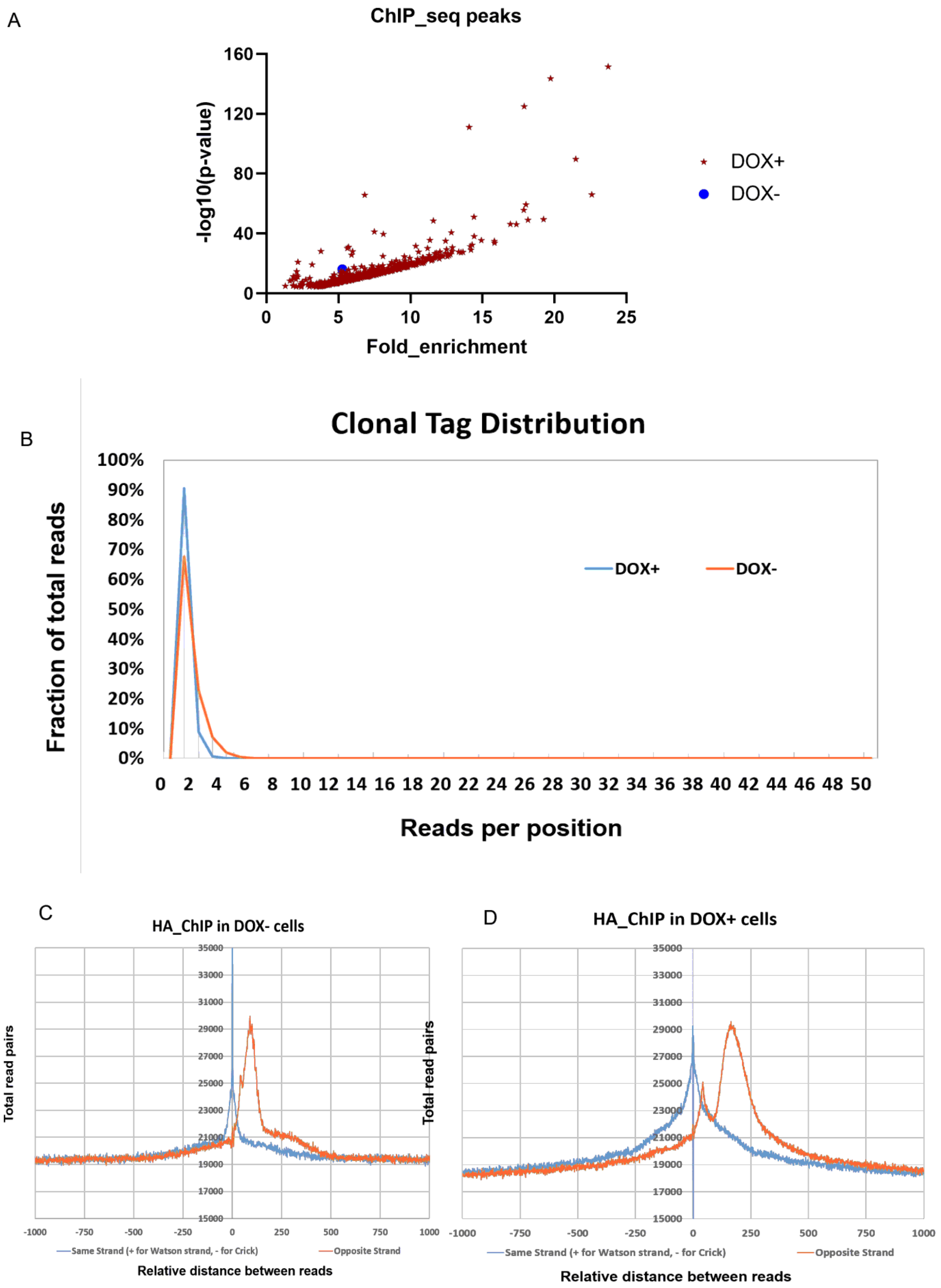
A
ChIP_seq peaks
-log10(p-value)
Fold_enrichment
DOX+
DOX-
B
Clonal Tag Distribution
Fraction of total reads
Reads per position
DOX+
DOX-
C
HA_ChIP in DOX- cells
Total read pairs
Same Strand (+ for Watson strand, - for Crick)
Opposite Strand
Relative distance between reads
D
HA_ChIP in DOX+ cells
Total read pairs
Same Strand (+ for Watson strand, - for Crick)
Opposite Strand
Relative distance between reads

Figure S11: ChIP-Seq QC related to STAR methods

A) Different peak distributions in Dox+ and Dox- all significant (FDR < 0.05) MACS2 output peaks.

B) Tag distribution analyzed by Homer showing the number of reads per unique position, which shows that experiment was not over-sequenced.

C) Autocorrelation analyzed by Homer in Dox+ cells shows the distribution of distances between adjacent reads in the genome based on which strand they map to in the genome. Average fragment length is around 200bp.

D) Autocorrelation analyzed by Homer in Dox- cells.

References

1. Yan, L., Yang, M., Guo, H., Yang, L., Wu, J., Li, R., Liu, P., Lian, Y., Zheng, X., Yan, J., Huang, J., Li, M., Wu, X., Wen, L., Lao, K., Li, R., Qiao, J., & Tang, F. (2013). Single-cell RNA-Seq profiling of human preimplantation embryos and embryonic stem cells. *Nature Structural and Molecular Biology*, *20*(9), 1131–1139. https://doi.org/10.1038/nsmb.2660
2. Medvedeva, Y. A., Lennartsson, A., Ehsani, R., Kulakovskiy, I. V, Vorontsov, I. E., Panahandeh, P., Khimulya, G., Kasukawa, T., Consortium, T. F., & Drabløs, F. (2015). EpiFactors: a comprehensive database of human epigenetic factors and complexes. *Database*, *2015*, bav067. https://doi.org/10.1093/database/bav067
3. Uhlen, M., Zhang, C., Lee, S., Sjöstedt, E., Fagerberg, L., Bidkhori, G., Benfeitas, R., Arif, M., Liu, Z., Edfors, F., Sanli, K., Von Feilitzen, K., Oksvold, P., Lundberg, E., Hober, S., Nilsson, P., Mattsson, J., Schwenk, J. M., Brunnström, H., … Ponten, F. (2017). A pathology atlas of the human cancer transcriptome. *Science*, *357*(6352). https://doi.org/10.1126/science.aan2507
4. Vuoristo, S., Hydén-Granskog, C., Yoshihara, M., Bhagat, S., Gawriyski, L., Jouhilahti, E.-M., Damdimopoulos, A., Ranga, V., Tamirat, M., Huhtala, M., et al. (2020). DUX4 regulates oocyte to embryo transition in human. bioRxiv, 732289. 10.1101/732289.
5. Balboa, D., Weltner, J., Eurola, S., Trokovic, R., Wartiovaara, K., & Otonkoski, T. (2015). Conditionally Stabilized dCas9 Activator for Controlling Gene Expression in Human Cell Reprogramming and Differentiation. *Stem Cell Reports*, *5*(3), 448–459. https://doi.org/https://doi.org/10.1016/j.stemcr.2015.08.001
6. Wu, J., Xu, J., Liu, B., Yao, G., Wang, P., Lin, Z., Huang, B., Wang, X., Li, T., Shi, S., Zhang, N., Duan, F., Ming, J., Zhang, X., Niu, W., Song, W., Jin, H., Guo, Y., Dai, S., … Sun, Y. (2018). Chromatin analysis in human early development reveals epigenetic transition during ZGA. *Nature*, *557*(7704), 256–260. https://doi.org/10.1038/s41586-018-0080-8

7. Liu, L., Leng, L., Liu, C., Lu, C., Yuan, Y., Wu, L., Gong, F., Zhang, S., Wei, X., Wang, M., Zhao, L., Hu, L., Wang, J., Yang, H., Zhu, S., Chen, F., Lu, G., Shang, Z., & Lin, G. (2019). An integrated chromatin accessibility and transcriptome landscape of human pre-implantation embryos. *Nature Communications*, *10*(1), 364. https://doi.org/10.1038/s41467-018-08244-0
8. Jouhilahti, E. M., Madissoon, E., Vesterlund, L., Töhönen, V., Krjutškov, K., Reyes, A. P., Petropoulos, S., Månsson, R., Linnarsson, S., Buörglin, T., Lanner, F., Hovatta, O., Katayama, S., & Kere, U. (2016). The human PRD-like homeobox gene LEUTX has a central role in embryo genome activation. *Development (Cambridge)*, *143*(19), 3459–3469. https://doi.org/10.1242/dev.134510
